# Trend and projection of mortality rate due to non-communicable diseases in Iran: A modeling study

**DOI:** 10.1371/journal.pone.0211622

**Published:** 2019-02-14

**Authors:** Fatemeh Khosravi Shadmani, Farshad Farzadfar, Bagher Larijani, Moghadameh Mirzaei, Ali Akbar Haghdoost

**Affiliations:** 1 Modeling in Health Research Center, Institute for Futures Studies in Health, Kerman University of Medical Sciences, Kerman, Iran; 2 Non-Communicable Diseases Research Center, Endocrinology and Metabolism Population Sciences Institute, Tehran University of Medical Sciences, Tehran, Iran; 3 Endocrinology and Metabolism Clinical Sciences Institute of Tehran University of medical sciences, Tehran, Iran; 4 Department of biostatistics and epidemiology, School of Public Health, Kerman University of Medical Sciences, Kerman, Iran; 5 Regional Knowledge Hub, and WHO Collaborating Centre for HIV Surveillance, Institute for Futures Studies in Health, Kerman University of Medical Sciences, Kerman, Iran; The University of Warwick, UNITED KINGDOM

## Abstract

**Background:**

Following the epidemiologic and demographic transition, non-communicable disease mortality is the leading cause of death in Iran. Projecting mortality trend can provide valuable tools for policy makers and planners. In this article, we have estimated the trend of non-communicable disease mortality during 2001–2015 and have projected it until 2030 at national and subnational levels in Iran.

**Methods:**

The data employed was gathered from the Iranian death registration system and using the Spatio-temporal model, the trends of 4 major categories of non-communicable diseases (cancers, cardiovascular diseases, asthma and COPD, and diabetes) by 2030 were projected at the national and subnational levels.

**Results:**

The results indicated that age standardized mortality rate for cancers, CVDs, and Asthma and COPD will continue to decrease in both sexes (cancers: from 81.8 in 2015 to 45.2 in 2030, CVDs: 307.3 to 173.0, and Asthma and COPD: from 52.1 to 46.6); however, in terms of diabetes, there is a steady trend in both sexes at national level (from 16.6 to 16.5). Age standardized mortality rates for cancers and CVDs, in males and females, were high in all provinces in 2001. The variation between the provinces is clearer in 2015, and it is expected to significantly decrease in all provinces by 2030.

**Conclusion:**

Generally, the age standardized mortality rate from NCDs will decrease by 2030. Of course, given the experience of the past two decades in Iran, believing that the mortality rate will decrease may not be an easy notion to understand. However hard to believe, this decrease may be the result of better management of risk factors and early detection of patients due to more comprehensive care in all segments of society, as well as improved literacy and awareness across the country.

## Introduction

Due to the epidemiologic and demographic transition around the world, paying attention to non-communicable diseases (NCDs) is now considered a priority. As a result of prioritizing NCDs, target 3–4 of the sustainable development goals (SDGs) was introduced to reduce the total NCDs mortality rate by one third by 2030 [1].

NCDs claimed 71.3% (70.8–72.0) of all deaths worldwide in 2016 (5). Also, the disability-adjusted life year (DALYs) for NCDs was reported to be 59.7 (61.7–7.7%) in the same year [2]. It should be taken into consideration that the NCDs mortality and burden are distributed unequally over the world. It is reported that 80% of all NCDs related deaths occur in low and middle-income countries [3]. NCDs in Iran, a middle income country, account for 79.2% (77.7% -80.7%) of all deaths and 74% (71.5–76.4%) of the burden of diseases [2].

NCDs related deaths impose a huge burden upon communities and health care systems [4]. The complexity comes up when the pace of health transition is faster than the pace of development in health services. Therefore, policymakers and health planners need to have a deep understanding of the needs and priorities in order to embark on implementing optimal allocation of resources and providing appropriate service packages [5]. Estimating the mortality pattern in the course of time and predicting its status in the future can provide valuable tools for delivering the data needed by policy makers and planners.

For the time being, there are no national and subnational studies in Iran concerning the projection of deaths from NCDs. The aim of this study was to determine the trend and to project mortality rate from NCDs by 2030. This study considers the health gap in the Iranian population and its differences among the provinces, which can be used as a guide for evidence-based policy-making at the national and provincial levels.

## Methods

According to the data gathered through Iranian Death Registry System (DRS) and by using the Spatio-temporal model, the trends of 4 major categories of NCDs (cancers, cardiovascular diseases, asthma and COPD, and diabetes) were projected at the national and subnational levels by 2030.

### Data sources

#### Death data

Since 1995 all deaths in Iran have been registered via death certificate. Hospitals account for more than 60% of total number of deaths. Other deaths that occur outside the hospital (e.g. in private and public clinics) first must be approved by the physician and then death certificate would be issued. Abnormal and suspicious deaths are referred to the forensic medicine to determine the exact cause of death. Also, for the home deaths, if the cause of death is natural, the death certificate is issued by the physician. Without death certificate, the permission of burial in the cemetery will not be granted.

The task of collecting reports and controlling its quality from various sources (hospital, Maternity hospital, urban and rural health centers, forensic medicine, clinic, etc.) is the responsibility of the city health center. If the information is of good quality, then it will be registered in the death registration software; however, if the information contained in the death certificate is not sufficiently qualified or if there are garbage codes, or the causes are ambiguous or unlikely, then the information would be referred to the physician in order to be modified. Data recording in the death registration software is performed by the trained person who knows how the information is entered into the software and has the ability to code and determine the underlying cause of death in accordance with ICD-10 rules. After registering the deceased's information in the death registration software, the information is sent to the online integrated national death system. Then repeated records will be deleted.

**Fig 1:**
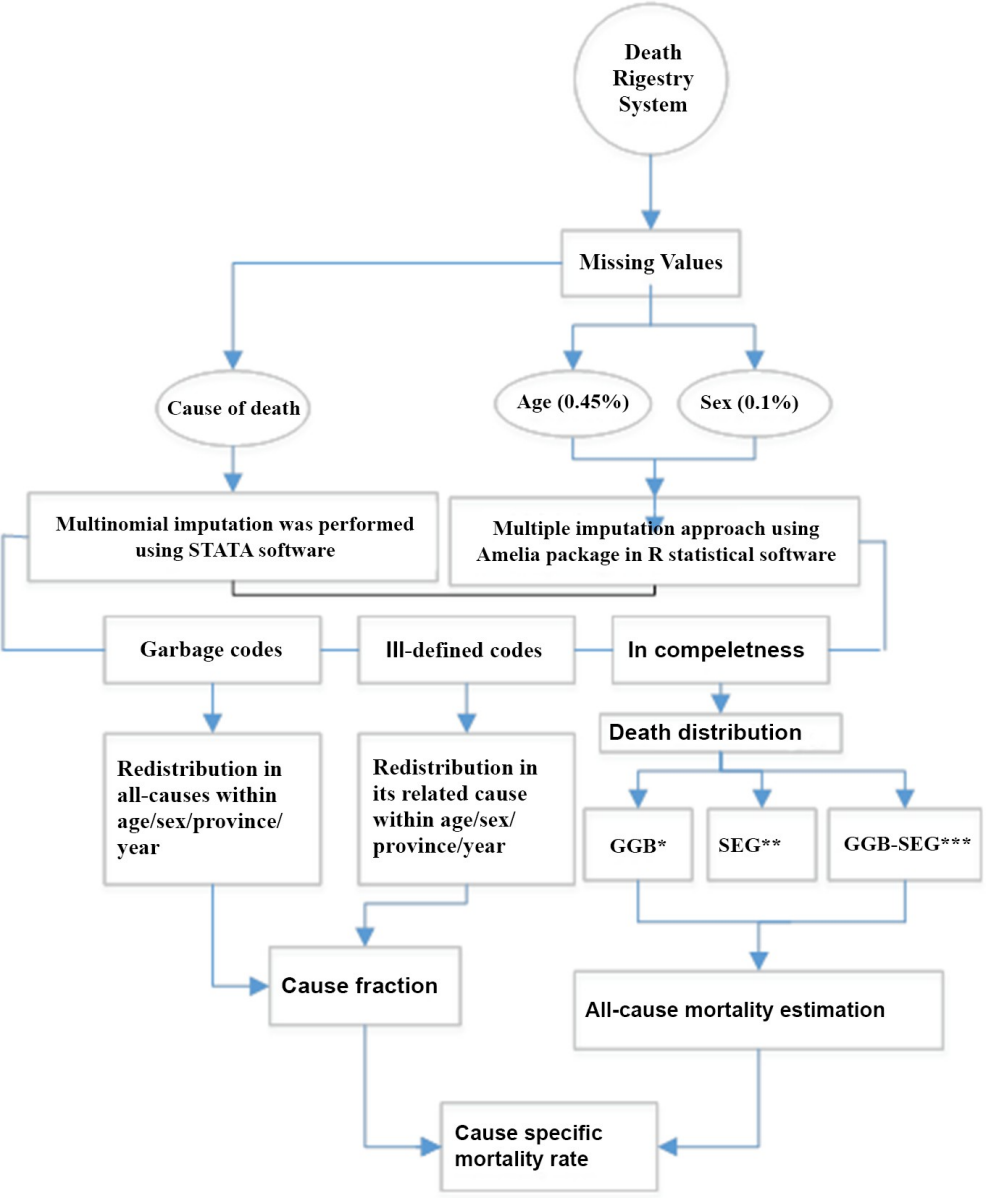
Flowchart for death redistribution. * Generalized Growth Balance. **Synthetic Extinct Generation. *** Generalized Growth Balance- Synthetic Extinct Generation.

In the Death Registry System, there were some instances of incompleteness and misclassification that were addressed through demographic and statistical methods. Redistribution method was used to correct the misclassification in the cause of death and the inconsistency of the cause with age and sex (Fig 1). Missing in cause of death and Garbage, and ill-defined codes were estimated to be 13.2% in 2015, more details are available elsewhere (6). There were several types of duplicate data in the recorded deaths that were corrected. Also, over the years, due to the changes in political divisions, the number of the provinces in Iran have been increased. As of 2011, there were some 31 provinces in Iran; however, in 2001, the total number of provinces in Iran was 28. Therefore, the data was constructed for the newly established provinces [6]. The Generalized Linear Mixed Model, Gaussian Process Regression (GPR) and age-spatio- temporal were employed in order to estimate all-cause mortality rates [7]. The mixed model residuals were evaluated according to age, time and space. The evaluated residuals, then, were added to the previous estimates. At the end, in order to estimate uncertainty interval for each point estimate, GPR modeling was employed [6].

Data gathered from 1995 to 2000 recorded the causes of death only in 17 major categories. However, since 2001 the death registration system became more detailed, recording all causes of death. Due to the little consistency between the data gathered before and after 2001 and validity of the data, this study did not use the data gathered before 2001. Tehran and Isfahan related data were not available for some years; therefore, information was collected from the two cemeteries of Behest-e Zahra in Tehran and Bagh-e Rezvan in Isfahan. Detailed description has been included elsewhere [6].

#### Population data

Calculating mortality rate required estimating high-risk population in the middle of each year both for sexes and provinces. Demographic data was extracted from censuses conducted once every 5 years in Iran. The growth model was also used to estimate the population over the 5-year period.

#### Covariate data

Households Income and Expenditure Survey (HIES) was started in 1963 in Iranian rural areas. Since 1968 this survey has been conducted in Iranian urban areas aiming at assessing the socioeconomic situation and estimating the average income and household expenses. The data on the variables of the wealth index, years of schooling, and urbanization until 2015 by age, sex, and province of residence were derived from this survey. Also, wealth index, years of schooling, and urbanization covariates until 2030 were estimated using the spline method and then entered into the covariate model.

### Statistical analysis

NCDs deaths were extracted from the Iranian death registration system based on the ICD10 codes (I00 to I99, C00 to C97, E10 to E14, J30 to J98) by age, sex, cause, province of residence, and year. Deaths from 2001 to 2015 were categorized into 13 age groups with 5-year intervals (25–29, 30–34, …, 80–84, 85+) and four categories of cancers, cardiovascular diseases, asthma and COPD, and diabetes. Mortality rate in each of the four categories was estimated through dividing the number of deaths for age, sex, province of residence by the population.

We used Iranian population in 2015 as the standard population for standardization. Standardization was conducted with direct method in order to compare different provinces in terms of age. Then the age standardized mortality rate trend was drafted for the studied years. Since age standardized mortality rate of the provinces are in direct correlation with time, the usual models did not meet the expectations; therefore, spatio-temporal model was employed to make projection.

There were some instances of random effects of spatial units and a few fixed effects of covariates (urbanization, years of schooling, and wealth index) in the mixed model. Spatio-temporal model was brought into R version 3.4.2 (28-09-2017) software, and to obtain the model parameters, the Markov Chain Monte Carlo (MCMC) simulation algorithm was used with 10,000 iterations.

## Results

**Fig 2:**
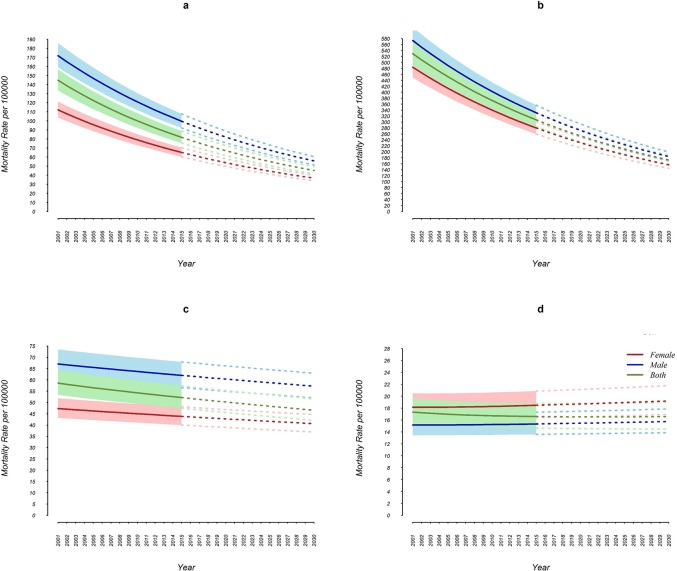
Trend and projection of age standardized mortality rate per 100000 by cause. a) Cancers, b) CVDs, c) Asthma and COPD, d) Diabetes.

The results indicated that age standardized mortality rate for cancers, CVDs and asthma, and COPD will decrease in males, females and both sexes combined in the course of 2015 to 2030 (Fig 2). However, diabetes in males and females will increase substantially whereas the trend of this condition in both sexes will remain steady at national level (Fig 2). The amount of this reduction is not the same for the 3 categories; the percent change in age standardized mortality rate from cancer is expected to be -44.7 (age standardized mortality rate would decrease from 81.8 (75.5–88.7) in 2015 to 45.2 (41.4–49.4) in 2030) (Table 1). Furthermore, the percent change in age standardized mortality rate from CVDs is expected to be -43.6 (age standardized mortality rate would decrease from 307.3 (284.2–332.4) to 173.0 (158.5–189.0)) (Table 1). Also, the percent change in age standardized mortality rate from asthma and COPD is expected to be -10.6 (age standardized mortality rate would decrease from 52.1 in 2015 (47.4–57.3) to 46.6 (42.0–51.6) in 2030) (Table 1). However, age standardized mortality rate from diabetes in both sexes would be steady during the same period of time (from 16.6 (14.6–18.8) to 16.5(14.4–18.9), indicating zero percent change) (Table 1). At subnational level, Zanjan and Hormozgan provinces, respectively, will have the highest and lowest percent change from cancers and CVDs, (Table 1). Zanjan and Semnan provinces, respectively, will have the highest and lowest percent change for asthma and COPD and finally, Zanjan and Chaharmahal &Bakhtiari provinces, respectively, will have the highest and lowest percent change for diabetes (Table 1).

**Table 1:** Age-standardized mortality rates and percent change (from 2015 to 2030) for NCDs in both sexes for provinces in Iran, by cause.

	Cancers				Cardiovascular diseases				Ashtma & COPD				Diabetes			
Provinces	2001	2015	2030	% Change*	2001	2015	2030	% change	2001	2015	2030	% change	2001	2015	2030	% change
**Iran**	**144.8**	**81.8**	**45.2**	**-44.7**	**529.7**	**307.3**	**173.0**	**-43.6**	**58.6**	**52.1**	**46.6**	**-10.6**	**17.3**	**16.6**	**16.5**	**0.0**
	(129.3–150.8)^**$**^	(78.4–91.5(	(46.0–54.2)		(484.9–562.2)	(288.1–334.1)	(165.1–193.6)		(51.9–62.3)	(49.2–59.1)	(46.5–56.3)		(14.3–18.2)	(15.6–19.9)	(17.3–22.3)	
Markazi	139.7	84.7	49.9	-41.0	522.1	310.3	178.8	-42.3	56.9	53.9	51.2	-5.1	16.1	17.6	19.6	11.4
	(129.3–150.8)	(78.4–91.5)	(46.0–54.2)		(484.9–562.2)	(288.1–334.1)	(165.1–193.6)		(51.9–62.3)	(49.2–59.1)	(46.5–56.3)		(14.3–18.2)	(15.6–19.9)	(17.3–22.3)	
Gilan	150.9	86.6	48.1	-44.4	549.5	322.6	182.6	-43.3	60.0	53.7	48.0	-10.6	18.5	18.1	17.8	-1.5
	(139.1–163.6)	(79.7–94.1)	(43.9–52.5)		(507.9–594.5)	(297.5–349.9)	(167.1–199.6)		(50.1–60.5)	(43.4–53.0)	(37.2–46.4)		(13.3–17.1)	(12.0–15.7)	(10.8–14.6)	
Mazandaran	135.4	75.0	40.0	-46.6	522.5	302.9	169.2	-44.1	55.0	48.0	41.5	-13.4	15.1	13.8	12.6	-8.6
	(124.8–146.8)	(68.8–81.8)	(36.3–44.1)		(482.9–565.5)	(278.1–329.9)	(153.3–186.7)		(50.1–60.5)	(43.4–53.0)	(37.2–46.4)		(13.3–17.1)	(12.0–15.7)	(10.8–14.6)	
East Azerbaijan	147.3	78.9	41.3	-47.5	530.8	301.1	167.1	-44.4	59.5	50.8	43.3	-14.6	17.9	15.3	13.5	-11.7
	(136.2–159.2)	(73.0–85.2)	(38.0–44.9)		(492.3–572.3)	(279.6–324.4)	(154.0–181.3)		(54.3–65.3)	(46.3–55.6)	(39.3–47.7)		(15.8–20.2)	(13.6–17.3)	(11.9–15.4)	
West Azerbaijan	145.3	72.3	34.6	-52.1	524.1	286.5	151.3	-47.1	59.5	47.9	38.2	-20.1	17.6	13.1	9.8	-25.4
	(134.3–157.2)	(66.7–78.3)	(31.6–37.8)		(485.7–565.5)	(265.3–309.4)	(138.5–165.3)		(54.2–65.3)	(43.6–52.6)	(34.5–42.4)		(15.5–19.8)	(11.6–14.8)	(8.5–11.2)	
Kermanshah	154.2	85.2	45.4	-46.6	541.6	310.2	171.9	-44.5	61.8	54.3	47.3	-12.7	19.5	17.8	16.3	-8.2
	(142.5–166.9)	(78.8–92.0)	(41.9–49.2)		(501.8–584.6)	(287.9–334.1)	(159.1–185.6)		(56.4–67.9)	(49.5–59.5)	(43.1–52.0)		(17.3–22.1)	(15.8–20.1)	(14.4–18.5)	
Khuzestan	135.9	80.9	48.0	-40.7	510.2	303.4	177.9	-41.3	55.8	51.9	49.0	-5.7	15.3	16.1	18.0	11.6
	(142.5–166.9)	(78.8–92.0)	(41.9–49.2)		(472.5–551.1)	(281.5–327.0)	(164.4–192.6)		(50.9–61.3)	(47.4–56.9)	(44.5–53.9)		(13.6–17.4)	(14.3–18.2)	(15.9–20.4)	
Fars	143.4	86.8	51.5	-40.5	531.1	317.8	185.6	-41.6	57.6	54.3	51.6	-5.1	16.8	18.3	20.6	12.4
	(132.7–154.9)	(80.3–93.9)	(47.3–56.1)		(493.3–571.9)	(294.8–342.6)	(170.7–201.7)		(52.6–63.1)	(49.6–59.6)	(46.7–56.9)		(14.9–19.0)	(16.2–20.7)	(18.1–23.5)	
Kerman	144.8	77.1	39.1	-49.1	531.2	301.0	164.1	-45.4	58.6	49.8	41.7	-16.1	17.3	14.7	12.3	-16.4
	(134.0–156.4)	(71.2–83.5)	(35.7–42.9)		(493.3–572.1)	(278.7–325.1)	(149.8–179.8)		(53.4–64.1)	(45.4–54.7)	(37.6–46.4)		(15.3–19.5)	(13.0–16.6)	(10.7–14.1)	
Razavi Khorasan	144.1	81.8	44.5	-45.5	529.2	306.3	170.6	-44.2	58.1	52.2	46.6	-10.8	17.1	16.4	15.8	-4.1
	(133.4–155.6)	(75.7–88.4)	(41.1–48.3)		(491.4–570.0)	(284.4–329.8)	(157.9–184.3)		(53.0–63.7)	(47.7–57.2)	(42.4–51.1)		(15.1–19.2)	(14.6–18.5)	(13.9–17.8)	
Isfahan	143.5	82.7	45.6	-44.8	532.8	307.2	169.8	-44.7	57.0	52.4	47.8	-8.8	16.7	16.7	16.5	-0.9
	(132.4–155.6)	(76.3–89.6)	(41.9–49.6)		(492.6–576.2)	(283.9–332.3)	(156.4–184.3)		(51.8–62.6)	(47.6–57.6)	(43.3–52.6)		(14.7–18.9)	(14.7–18.9)	(14.5–18.8)	
Sistan and Baluchistan	161.1	83.1	41.3	-50.2	548.3	303.4	162.1	-46.5	65.3	54.5	45.2	-17.1	21.6	17.3	14.0	-19.3
	(148.2–175.2)	(76.7–90.1)	(38.0–44.9)		(505.2–595.1)	(280.5–328.1)	(149.5–175.9)		(59.2–72.0	(49.6–59.9)	(41.0–49.8)		(18.9–24.5)	(15.3–19.7)	(12.3–15.9)	
Kurdistan	147.6	70.0	32.0	-54.3	524.7	281.3	146.0	-48.0	60.6	46.9	35.9	-23.4	18.2	12.4	8.4	-32.0
	(136.2–159.9)	(64.6–76.0)	(29.0–35.2)		(485.3–567.3)	(260.0–304.4)	(132.7–160.7)		(55.2–66.6)	(42.7–51.6)	(32.2–40.0)		(16.0–20.6)	(10.9–14.0)	(7.30–9.7)	
Hamadan	141.1	82.3	46.9	-43.0	522.0	308.4	177.7	-42.3	57.7	52.6	48.0	-8.8	16.5	16.7	17.2	3.2
	(130.6–152.5)	(76.2–89.0)	(43.1–51.0)		(484.5–562.4)	(286.3–332.2)	(163.6–192.9)		(52.7–63.3)	(48.0–57.7)	(43.5–52.9)		(14.6–18.6)	(14.8–18.8)	(15.1–19.6)	
Chaharmahal and Bakhtiari	147.1	84.3	47.0	-44.2	532.7	311.7	177.3	-43.1	59.5	53.7	48.4	-9.8	17.9	17.5	17.4	-0.3
	(136.1–159.0)	(78.0–91.1)	(43.2–51.1)		(494.5–573.9)	(289.3–335.9)	(163.3–192.5)		(54.3–65.2)	(49.0–58.8)	(43.9–53.3)		(15.8–20.1)	(15.5–19.7)	(15.3–19.8)	
Lorestan	150.8	74.8	35.6	-52.3	534.6	293.4	155.3	-47.0	61.1	49.0	38.8	-20.7	18.8	13.9	10.3	-26.3
	(139.3–163.1)	(69.2–80.9)	(32.6–38.9)		(495.5–576.8)	(272.1–316.3)	(142.2–169.6)		(55.7–67.0)	(44.7–53.7)	(35.0–43.0)		(16.6–21.2)	(12.3–15.7)	(8.9–11.8)	
Ilam	146.2	84.5	47.5	-43.7	529.1	312.0	178.7	-42.7	59.5	53.7	48.4	-9.8	17.7	17.5	17.6	0.7
	(135.2–158.0)	(78.3–91.3)	(43.7–51.6)		(491.0–570.3)	(289.7–336.0)	(164.7–193.8)		(54.2–65.2)	(49.0–58.8)	(43.9–53.4)		(15.7–20.0)	(15.0–19.8)	(15.5–20.1)	
Kohgiluyeh and Boyer_Ahmad	148.3	77.6	38.7	-50.0	534.4	300.2	161.9	-46.0	60.5	50.4	41.5	-17.7	18.3	14.9	12.0	-19.4
	(136.7–160.9)	(71.6–84.0)	(35.5–42.2)		(493.6–578.7)	(277.9–324.4	(148.8–176.3)		(55.0–66.6)	(45.9–55.4)	(37.5–45.8)		(16.1–20.7)	(13.2–16.9)	(10.5–13.7)	
Bushehr	140.7	97.8	69.1	-29.3	508.5	333.6	194.2	-41.7	54.0	56.5	60.4	6.9	16.3	23.1	35.6	54.1
	(132.1–149.7)	(91.4–104.7)	(63.0–75.8)		(478.5–540.4)	(235.3–285.1)	(177.5–212.5)		(50.1–58.2)	(52.2–61.2)	(54.4–67.1)		(14.4–18.3)	(20.4–26.1)	(30.5–41.4)	
Zanjan	135.3	56.5	23.0	-59.2	508.9	259.0	129.3	-50.0	56.2	39.0	26.9	-30.8	15.3	8.1	4.4	-45.7
	(125.1–146.4)	(51.3–62.1)	(20.1–26.3)		(471.7–549.0)	(235.3–285.1)	(112.3–148.8)		(51.2–61.6)	(35.0–43.4)	(23.2–31.2)		(13.6–17.3)	(7.0–9.4)	(3.6–5.4)	
Semnan	136.9	86.2	52.6	-38.9	521.6	319.9	190.4	-40.4	55.2	53.3	51.4	-3.6	15.4	17.9	21.1	17.8
	(126.6–148.0)	(79.5–93.3)	(48.0–57.6)		(484.1–562.1)	(296.0–345.7)	(173.8–208.5)		(50.4–60.5)	(48.6–58.6)	(46.3–57.1)		(13.6–17.3)	(15.8–20.2)	(18.3–24.2)	
Yazd	139.0	83.5	49.8	-40.3	521.3	314.0	186.4	-40.6	56.1	52.0	48.8	-6.1	15.9	16.8	18.9	12.2
	(128.5–150.5)	(77.0–90.4)	(45.4–54.6)		(483.0–562.8)	(290.6–339.4)	(170.0–204.4)		(51.1–61.6)	(47.4–57.2)	(43.9–54.3)		(14.0–17.9)	(14.8–19.0)	(16.4–21.7)	
Hormozgan	136.4	93.3	62.8	-32.7	513.7	325.3	200.6	-38.3	56.6	58.8	61.7	4.8	15.6	21.3	30.5	42.6
	(125.8–147.9)	(85.9–101.4)	(57.0–69.1)		(474.9–555.6)	(300.2–352.6)	(182.1–221.0)		(51.5–62.2)	(53.4–64.7)	(55.3–68.8)		(13.7–17.7)	(18.8–24.2)	(26.3–35.3)	
Tehran	160.5	93.3	51.1	-45.1	577.0	334.9	184.4	-44.9	60.2	55.7	50.7	-8.9	20.1	20.5	20.1	-1.7
	(146.5–175.8)	(85.3–102.0)	(46.8–55.8)		(526.8–632.1)	(306.5–365.9)	(169.0–201.1)		(54.2–66.8)	(50.3–61.7)	(45.8–56.1)		(17.5–23.2)	(17.8–23.5)	(17.6–23.0)	
Ardabil	145.5	77.2	39.7	-48.5	525.0	297.8	164.0	-44.9	59.6	50.2	42.0	-16.2	17.6	14.8	12.5	-15.0
	(134.4–157.4)	(71.4–83.4)	(36.5–43.2)		(486.6–566.5)	(276.5–320.8)	(151.0–178.3)		(54.3–65.4)	(45.8–55.0)	(38.1–46.4)		(15.6–19.9)	(13.1–16.7)	(11.0–14.3)	
Qom	136.2	77.3	43.6	-43.5	509.9	294.7	168.1	-42.9	55.4	49.9	45.5	-8.8	15.3	14.8	15.1	1.9
	(124.6–148.8)	(70.9–84.4)	(40.0–47.5)		(467.0–556.7)	(270.4–321.1)	(154.4–182.9)		(50.0–61.3)	(45.2–55.2)	(41.2–50.3)		(13.3–17.5)	(12.9–16.9)	(13.2–17.2)	
Qazvin	141.0	81.7	44.6	-45.3	523.5	303.5	167.2	-44.9	57.3	52.7	47.6	-9.6	16.4	16.5	16.1	-2.7
	(130.6–152.3)	(75.6–88.3)	(41.2–48.4)		(486.2–563.7)	(281.8–327.0)	(154.6–180.9)		(52.3–62.7)	(48.1–57.7)	(43.3–52.3)		(14.5–18.5)	(14.6–18.7)	(14.2–18.2)	
Golestan	140.6	87.7	53.3	-39.2	524.2	320.0	189.7	-40.7	57.5	55.2	53.1	-3.8	16.4	18.8	22.0	17.0
	(130.0–152.1)	(80.9–95.1)	(48.7–58.4)		(485.8–565.6)	(295.9–346.2)	(173.3–207.8)		(52.4–62.7)	(50.2–60.7)	(47.8–58.9)		(14.5–18.5)	(16.6–21.3)	(19.1–25.3)	
North Khorasan	142.9	75.9	38.7	-48.9	524.2	297.7	163.2	-45.1	58.8	49.5	41.2	-16.6	17.0	14.3	12.0	-16.3
	(131.9–154.8)	(70.0–82.2)	(35.4–42.3)		(485.0–566.5)	(275.4–321.9)	(149.3–178.4)		(53.5–64.5)	(45.0–54.4)	(37.2–45.7)		(15.0–19.3)	(12.6–16.2)	(10.4–13.7)	
South Khorasan	151.0	86.7	47.5	-45.2	538.2	318.1	180.6	-43.2	61.4	54.7	48.2	-11.9	18.9	18.4	17.61	-4.2
	(139.3–163.7)	(79.9–94.0)	(43.4–51.9)		(497.7–582.1)	(293.9–344.4)	(165.1–197.6)		(55.8–67.5)	(49.8–60.2)	(43.5–53.5)		(16.7–21.4)	(16.2–20.8)	(15.3–20.2)	
Alborz	151.4	91.7	52.9	-42.2	550.3	329.0	188.0	-42.8	58.8	55.6	52.0	-6.5	18.4	20.0	21.4	7.2
	(139.3–164.6)	(84.2–99.9)	(48.4–57.9)		(507.2–597.1)	(302.5–357.9)	(171.9–205.7)		(53.4–64.8)	(50.3–61.4)	(46.8–57.6)		(16.1–20.9)	(17.5–22.8)	(18.7–24.6)	

*percent change from 2015 to 2030

$ 95% confidence interval

**Fig 3:**
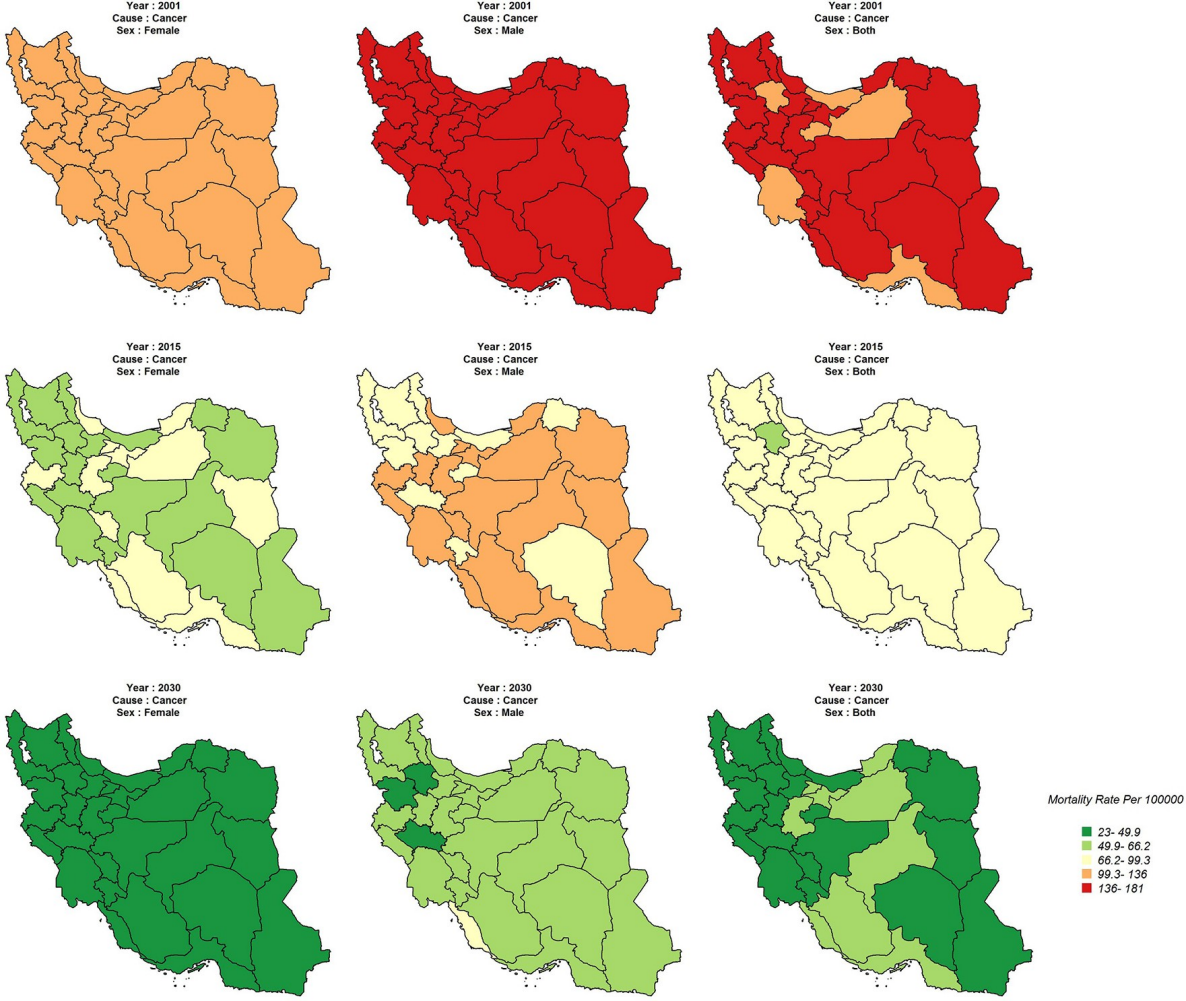
Age-standardized cancers mortality rate (Per 100,000) in 2001, 2015, 2030.

In general, the age standardized mortality rate for cancers, in males and females, was high for all provinces in 2001, hardly indicating any variation. However, the variation between provinces is quite clear in 2015. Age standardized mortality rate is expected to decrease significantly in each province by 2030 and there will hardly be any significant variation in females between the provinces. For both sexes, however, mortality rate will reduce more slowly in western, northern, and central provinces (Fig 3). Also, it is expected that the age standardized mortality rate for cardiovascular diseases is decreasing sharply for both sexes in all provinces by 2001 to 2030 (Fig 3).

**Fig 4:**
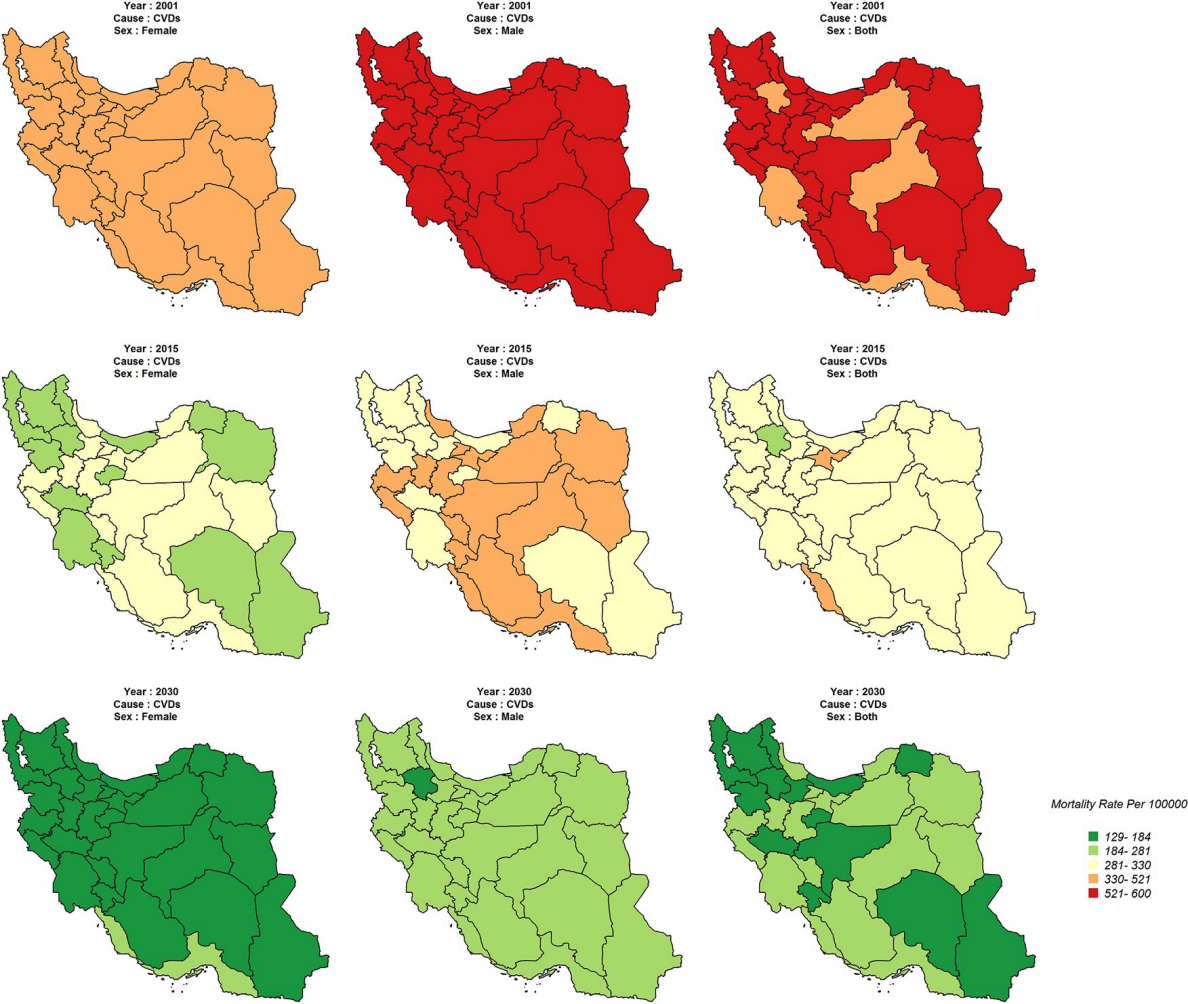
Age-standardized CVDs mortality rate (Per 100,000) in 2001, 2015, 2030.

Age standardized mortality rate for asthma and COPD, among males and females, was different in 2001, showing a higher rate in males than in females. However, this rate will decrease in some provinces over the course of time. Also, there will be provincial variations in males and in both sexes by 2030 (Fig 4).

**Fig 5:**
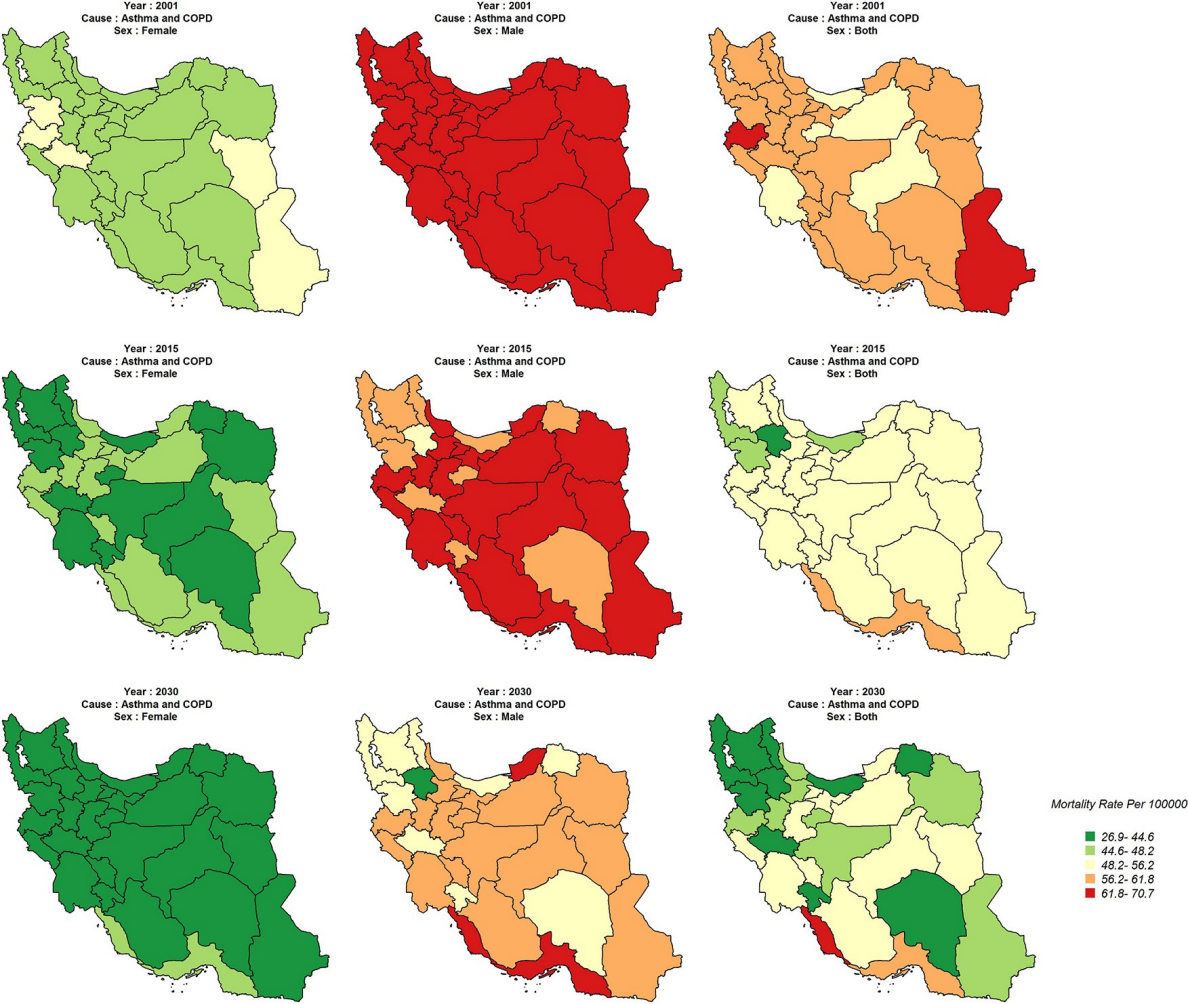
Age-standardized asthma&COPD mortality rate (Per 100,000) in 2001, 2015, 2030.

There will be no significant variation for diabetes between the provinces in 2001; whereas, the variation is expected to become more explicit in 2015. Also, the mortality rate will sharply decrease in southeastern and northwestern provinces (Fig 5).

**Fig 6:**
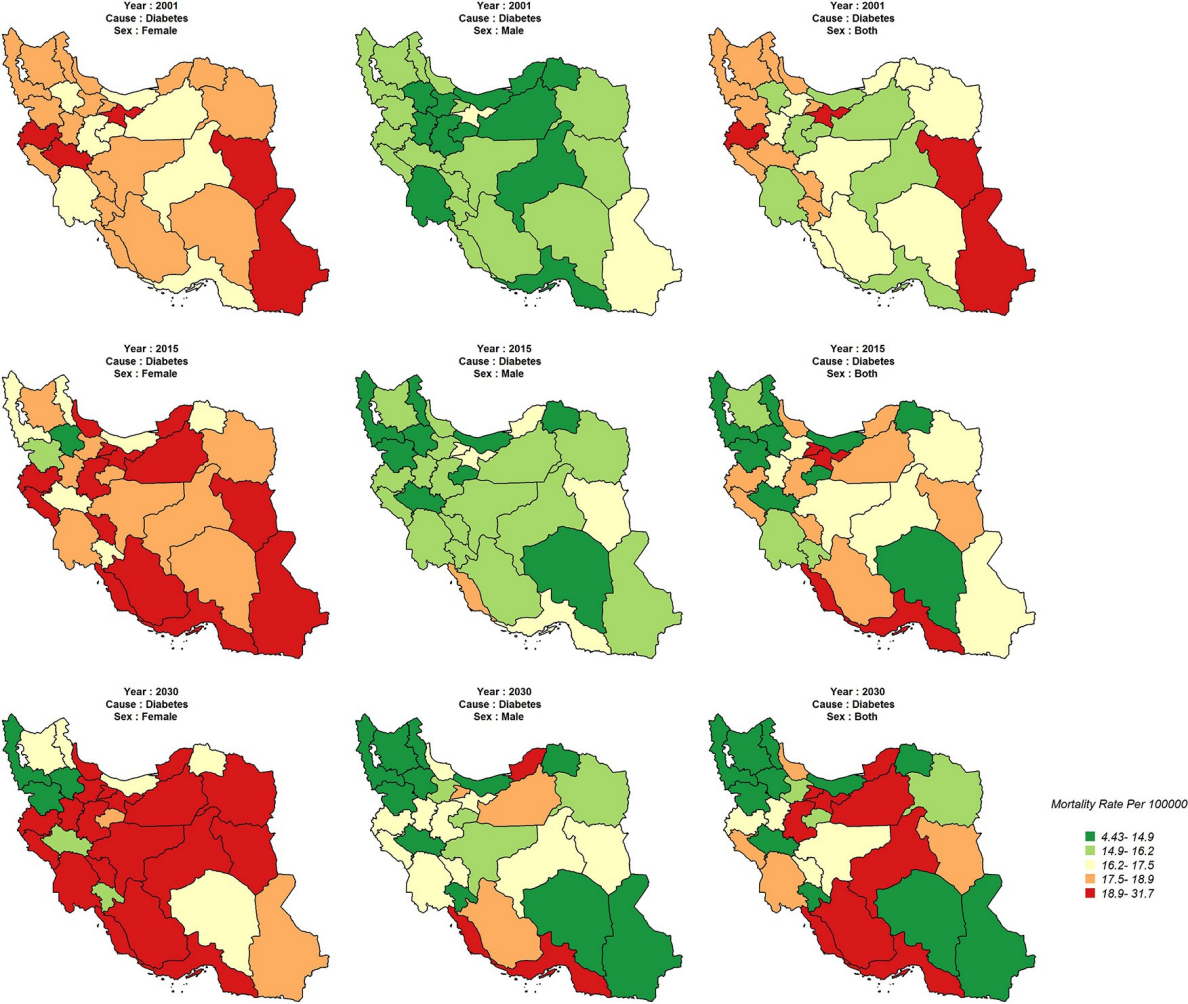
Age-standardized diabetes mortality rate (Per 100,000) in 2001, 2015, 2030.

The age standardized mortality rate for diabetes in 2001 was higher in females than in males, and is expected to increase in most provinces by 2030. However, this rate in female is expected to decrease in some southeastern provinces of Iran. The trend will almost be the same for males. It should be noted that there is a significant variation in both sexes for diabetes in all provinces (Fig 6). The trend of each of the 4 categories of NCDs by province is shown in appendix Fig 1.

**Fig 7:**
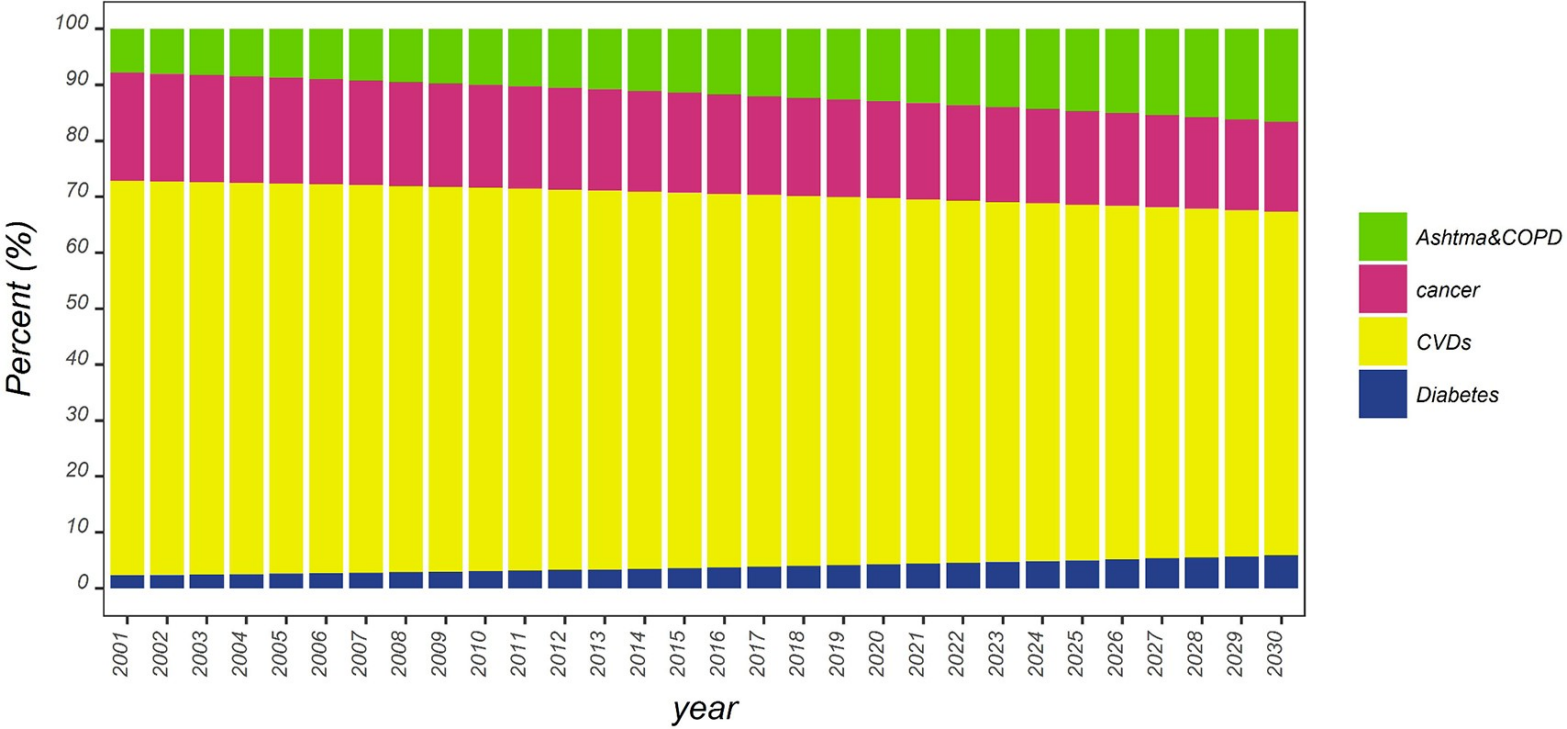
Proportional mortality from NCDs in Iran.

Generally, at the national level, about 70.5% of the proportional mortality of NCDs in 2001 was related to CVDs; in 2015, this indicator accounted for 67.1% of the proportional mortality and in 2030 it is expected to be 61.4%, showing a decreasing trend in the proportional mortality rate (Fig 7). The proportional mortality for cancers has also decreased from 19.2% in 2001 to 16.7% in 2015 and it is expected to drop to 16% by 2030 (Fig 7).

Proportional mortalities for asthma and COPD and diabetes show an increasing trend over time. The index for asthma and COPD in 2001, 2015, and 2030 is estimated to be 7.8, 11.3 and 16.5%, respectively (Fig 7). These numbers for diabetes are 2.3, 3.6 and 5.8 for 2001, 2015, and 2030, respectively (Fig 7). The pattern of proportional mortality in the provinces is similar to that of the whole country (Fig 7).

## Discussion

According to the results of this study, the trend of cancers and cardiovascular disease mortalities in Iran is expected to decrease moderately, while there will be a substantial decrease in asthma and COPD. Also, the trend of diabetes will slowly increase. The mortality rate of cancers, cardiovascular diseases, and asthma and COPD will be higher in males than in females, and this trend will continue until 2030. However, in terms of diabetes, the mortality rate will be higher in females and it will continue. At subnational level, cancers in the southern, northern, and central provinces will decrees slowly in both sexes; whereas, cardiovascular diseases in the southeastern, northwest, and central province will have a higher decrease in both sexes. The mortality rate for asthma and COPD will be higher in southern provinces and metropolitan areas. Mortality rate for diabetes in most provinces is high; whereas mortality rate will be even higher in the southern, central and Northern provinces. Also, cardiovascular diseases and diabetes have the highest and lowest proportional mortality rates respectively. At national and subnational levels, by 2030, proportional mortality trend for cardiovascular diseases is expected to decrease while this trend for diabetes and asthma and COPD will increase.

In a study carried out in Brazil to investigate the NCDs mortality during the period from 1990 to 2015, a decreasing trend was observed for cancers [8]. The results of a population-based cohort study conducted in Sweden to investigate the prevalence of NCDs premature deaths shows a 15.5 percent reduction in the cancers mortality rate during the period from 1991 to 2006. [9] In general, the pattern and trend of cancers in different countries depend on the inherent inequality and the growing gap between them in terms of health and medical infrastructure. Due to the success in controlling common cancers as a result of early screening and diagnosis, as well as effective prevention and treatment interventions, the incidence of cancers mortality in most developed countries is decreasing. However, cancers mortality rate in developing countries is steady or still on the rise [12–10] which is inconsistent with the results of the present study.

Some of studies have shown that cancers mortality in males is higher than females and males experience poor survival [13, 14]. This argument is in line with the results of this research. This difference in mortality rate between males and females can be the result of experiencing different risk factors including tobacco smoking, occupational exposure or hormonal changes.

As mentioned earlier, the mortality trend for cardiovascular diseases in Iran is higher in males but it will decrease moderately. These results are, to a large extent, consistent with the results of studies conducted in different countries. For instance, the estimates of heart disease from premature mortality, carried out in 188 countries in the period from 2013 to 2025, showed that the overall trend of premature mortality rate from cardiovascular diseases is decreasing. With the decrease in the prevalence of risk factors of cardiovascular diseases, it will be possible to achieve a 25% reduction in the mortality rate by 2025[15] Another study carried out in the United States showed that cancers and cardiovascular diseases mortality rates had been decreasing. It is also indicated that there was a decreasing trend with the total number of deaths falling from 175,000 to 135,000 among white males and females during the period from 2000 to 2014, respectively[16].

In another study conducted in Brazil to investigate the mortality trends over the course of 2001 to 2011, the results showed that despite an increase in the overall number of cardiovascular diseases related deaths, the age-adjusted mortality rate for these diseases was cut by 24% [17].

Also, cardiovascular disease mortality fell from 12.75 in 2000 to 10.09 in 2011 in Argentina [18].

In China, during the course of 1990 to 2013, mortality trend of ischemic heart disease in males had been increasing, where, it had been decreasing among females. Also, there were an increasing trend for cancers and a decreasing trend for COPD[19].

In general, various studies conducted on the myocardial infarction trend in the United States, Brazil, Japan, England, Sweden, Canada, Ireland, and Denmark show that the incidence of In- and Out-of-hospital mortality in these countries has been decreasing for the past two decades [24–20]

In a study conducted by Varmaghani et al., with the aim of investigating the trend of asthma and COPD mortality over the period from 2001 to 2015 in Iran, the results showed that the trend of COPD mortality had increased during this period and the age-standardized COPD mortality rate was higher in males (12.3) than in females (8.4). In the same study, the age-standardized mortality rate from asthma was also higher in males (8.8%) than in females (7.2%), and a decrease was observed in the overall trend of asthma[25].

Studies have shown that as the aging population grows, the prevalence of COPD and its related mortality rate increases[26, 27] Also, improved diagnoses may increase the COPD-specific mortality rate over time. Perhaps one of the reasons for substantial decreasing trend of asthma and COPD in Iran is the increase in the number of air pollution days per year in all provinces of Iran. For example, a study in Tehran showed that during the 2002 to 2012 period, air quality had been decreasing by 12% [28]. Also, various studies have shown that air pollution plays an important role in increasing the incidence and deaths from asthma and COPD[29, 30]. Therefore, inappropriate changes in air pollution and increased levels of it can be of the major causes of substantial decrease in number of deaths from asthma and COPD in Iran.

A study conducted in Brazil, based on the Global Burden of Disease Project, to investigate NCDs mortality during the course of 1990 to 2015; this study showed that during this period, mortality rate of diabetes was on the rise [8]. In another study carried out in China to investigate a one-third reduction in premature NCDs deaths by 2030, the results showed that despite 13.1% decrease in the number of deaths from premature NCDs, diabetes showed an increasing trend [16] In Brazil, mortality trend of diabetes was investigated from 1980 to 2012. The results showed that the mortality rate of diabetes had been sharply increased in both sexes; however, in the 12-year period (2003–2012) decreasing trend was observed in females, whereas, the trend in males was higher caused a convergence[31]. In another study, the mortality from diabetes in females was observed to be higher [32] which is consistent with the findings of present research. Generally, the incidence of diabetes shows an increasing trend in most countries. This increase in diabetes may, according to various studies, be due to overweight and obesity epidemics, food consumption patterns and the consumption of high calorie diets, urban development, and sedentary lifestyles caused by the development of modern transportation[33–37].

The geographical distributions of some common cancers and ischemic heart disease for some years in Iran are available; however, since these statistics are not thorough, comparing them with the results of the present study is not possible. Also, due to the differences in age groups and the separate investigation of asthma and COPD, the geographical distribution of asthma and COPD in the study conducted by Varmaghani et al., [25] was hardly consistent with the results of our study.

Proportional mortality rate from cancers and cardiovascular diseases in all provinces is expected to decrease. Considering that if proportional mortality for a cause decreases, the proportional mortality for the other causes must increase, the proportional mortality from asthma and COPD and diabetes (except for Zanjan province) is expected to increase.

With the increase in population aging, the number of deaths from NCDs is expected to increase. In this study, the age standardized mortality rate will eliminate this problem. Given the experience of the past two decades, believing that the number of deaths will decrease may not be an easy notion to understand.

One of the most important reasons behind the reduction of cardiovascular mortality rate in developed countries may be the focus of health systems on reducing the prevalence of cardiovascular disease risk factors that resulted in improving primary prevention. In Iran, however, the prevalence of these risk factors is increasing [38]; therefore, it cannot be considered the reason for decreasing number of deaths from cardiovascular diseases. Some of the possible explanations for this decrease in Iran might be improving the accuracy of death certificates, improving the registration system, and reducing garbage codes for cardiovascular diseases.

The other explanations for this decrease might be better management of risk factors and early detection of patients due to more comprehensive care in all segments of society, as well as improved literacy and awareness across the country.

When it comes to cardiovascular diseases, decrease in the number of deaths might be the result of improvement of drug strategies and diagnostic and therapeutic interventions such as using antihypertensive drugs, statins, aspirin, heparin, beta-blocker, streptokinase and captopril. Various studies have shown that consumption of these drugs in cardiovascular patients has significantly increased over the past few years [18, 21, 39]

In order for this decreasing trend to continue, it is recommended that more attention be paid to the interventions carried out at the primary prevention. Undoubtedly, educational interventions at community level and raising people's awareness on the risk factors of cardiovascular diseases along with interventions conducted to reduce these factors can play significant roles in improving the health of the population and reducing the number of cardiovascular deaths in the future.

There were some limitations in this study. All health registry data have had some problems including incompleteness, misclassification, and duplications that were mostly addressed. Although most of the issues were solved, the validity of future studies could directly be affected by the correction of DRS. Also data availability was restricted for some new provinces that were addressed by misalignment procedures. One of the strengths of this study was employing 15-year national data to make prediction.

## Conclusion

Based on the results, cancers and cardiovascular diseases are expected to continue to decrease until 2030. Also, asthma and COPD will decrease substantially; whereas, the diabetes trend will be on the rise. In general, the long path to reduce all four diseases is yet to be paved. It is recommended, therefore, that policymakers and planners, along with early detection and treatment, focus more on primary prevention and community education through mass media because these interventions will be more cost-effective.

## Supporting information

S1 Table Percent change (from 2001 to 2015) for NCDs in both sexes for provinces in Iran, by cause. (DOCX)

S1 Fig Trend of proportional mortality from all cause (2001–2015). (TIF)

S2 Fig Trend and projection of age standardized mortality rate per 100000 by cause. a) Cancer, b) CVDs, c) Asthma and COPD, d) Diabetes.Markazi province.
(TIF)

S3 Fig Trend and projection of age standardized mortality rate per 100000 by cause. a) Cancer, b) CVDs, c) Asthma and COPD, d) Diabetes. North Khorasan province.
(TIF)

S4 Fig Trend and projection of age standardized mortality rate per 100000 by cause. a) Cancer, b) CVDs, c) Asthma and COPD, d) Diabetes. Hamedan province.
(TIF)

S5 Fig Trend and projection of age standardized mortality rate per 100000 by cause. a) Cancer, b) CVDs, c) Asthma and COPD, d) Diabetes. Golestan province.
(TIF)

S6 Fig Trend and projection of age standardized mortality rate per 100000 by cause. a) Cancer, b) CVDs, c) Asthma and COPD, d) Diabetes. Lorestan province.
(TIF)

S7 Fig Trend and projection of age standardized mortality rate per 100000 by cause. a) Cancer, b) CVDs, c) Asthma and COPD, d) Diabetes. Kordestan province.
(TIF)

S8 Fig Trend and projection of age standardized mortality rate per 100000 by cause. a) Cancer, b) CVDs, c) Asthma and COPD, d) Diabetes. Kohgiluyeh and Boyer- Ahmad province.
(TIF)

S9 Fig Trend and projection of age standardized mortality rate per 100000 by cause. a) Cancer, b) CVDs, c) Asthma and COPD, d) Diabetes. Kermanshah province.
(TIF)

S10 Fig Trend and projection of age standardized mortality rate per 100000 by cause. a) Cancer, b) CVDs, c) Asthma and COPD, d) Diabetes. Kerman province.
(TIF)

S11 Fig Trend and projection of age standardized mortality rate per 100000 by cause. a) Cancer, b) CVDs, c) Asthma and COPD, d) Diabetes. Isfahan province.
(TIF)

S12 Fig Trend and projection of age standardized mortality rate per 100000 by cause. a) Cancer, b) CVDs, c) Asthma and COPD, d) Diabetes. Ilam province.
(TIF)

S13 Fig Trend and projection of age standardized mortality rate per 100000 by cause. a) Cancer, b) CVDs, c) Asthma and COPD, d) Diabetes. Hormozgan province.
(TIF)

S14 Fig Trend and projection of age standardized mortality rate per 100000 by cause. a) Cancer, b) CVDs, c) Asthma and COPD, d) Diabetes. Khuzestan province.
(TIF)

S15 Fig Trend and projection of age standardized mortality rate per 100000 by cause. a) Cancer, b) CVDs, c) Asthma and COPD, d) Diabetes. Gilan province.
(TIF)

S16 Fig Trend and projection of age standardized mortality rate per 100000 by cause. a) Cancer, b) CVDs, c) Asthma and COPD, d) Diabetes. Fars province.
(TIF)

S17 Fig Trend and projection of age standardized mortality rate per 100000 by cause. a) Cancer, b) CVDs, c) Asthma and COPD, d) Diabetes. East Azarbaijan province.
(TIF)

S18 Fig Trend and projection of age standardized mortality rate per 100000 by cause. a) Cancer, b) CVDs, c) Asthma and COPD, d) Diabetes. Chaharmahal and Bakhtiari province.
(TIF)

S19 Fig Trend and projection of age standardized mortality rate per 100000 by cause. a) Cancer, b) CVDs, c) Asthma and COPD, d) Diabetes. Ardebil province.
(TIF)

S20 Fig Trend and projection of age standardized mortality rate per 100000 by cause. a) Cancer, b) CVDs, c) Asthma and COPD, d) Diabetes. Alborz province.
(TIF)

S21 Fig Trend and projection of age standardized mortality rate per 100000 by cause. a) Cancer, b) CVDs, c) Asthma and COPD, d) Diabetes. Zanjan province.
(TIF)

S22 Fig Trend and projection of age standardized mortality rate per 100000 by cause. a) Cancer, b) CVDs, c) Asthma and COPD, d) Diabetes. Yazd province.
(TIF)

S23 Fig Trend and projection of age standardized mortality rate per 100000 by cause. a) Cancer, b) CVDs, c) Asthma and COPD, d) Diabetes. West Azarbiajan province.
(TIF)

S24 Fig Trend and projection of age standardized mortality rate per 100000 by cause. a) Cancer, b) CVDs, c) Asthma and COPD, d) Diabetes. Tehran province.
(TIF)

S25 Fig Trend and projection of age standardized mortality rate per 100000 by cause. a) Cancer, b) CVDs, c) Asthma and COPD, d) Diabetes. South Khorasan province.
(TIF)

S26 Fig Trend and projection of age standardized mortality rate per 100000 by cause. a) Cancer, b) CVDs, c) Asthma and COPD, d) Diabetes. Siatan and bluchestan province.
(TIF)

S27 Fig Trend and projection of age standardized mortality rate per 100000 by cause. a) Cancer, b) CVDs, c) Asthma and COPD, d) Diabetes. Semnan province.
(TIF)

S28 Fig Trend and projection of age standardized mortality rate per 100000 by cause. a) Cancer, b) CVDs, c) Asthma and COPD, d) Diabetes. Razavi Khorasan province.
(TIF)

S29 Fig Trend and projection of age standardized mortality rate per 100000 by cause. a) Cancer, b) CVDs, c) Asthma and COPD, d) Diabetes. Qom province.
(TIF)

S30 Fig Trend and projection of age standardized mortality rate per 100000 by cause. a) Cancer, b) CVDs, c) Asthma and COPD, d) Diabetes. Qazvin province.
(TIF)

S31 Fig Trend and projection of age standardized mortality rate per 100000 by cause. a) Cancer, b) CVDs, c) Asthma and COPD, d) Diabetes. Mazandaran province.
(TIF)

S32 Fig Trend and projection of age standardized mortality rate per 100000 by cause. a) Cancer, b) CVDs, c) Asthma and COPD, d) Diabetes. Bushehr province.
(TIF)

## References

[1] The United Nations. Sustainable Development Goals. [cited 2018 1/10]; Available from: http://www.un.org/sustainabledevelopment/health/.

[2] (IHME)., I.f.H.M.a.E., et al. 2015 [cited 2018 1/10]; Available from: http://vizhub.healthdata.org/gbd-compare.

[3] Abegunde D.O., et al, The burden and costs of chronic diseases in low-income and middle-income countries. The Lancet, 2007 370(9603): p. 1929–1938. 10.1016/S0140-6736(07)61696-1 18063029

[4] Kontis V., et al, Contribution of six risk factors to achieving the 25×25 non-communicable disease mortality reduction target: a modelling study. The Lancet, 2014 384(9941): p. 427–437. 10.1016/S0140-6736(14)60616-4 24797573

[5] Global, regional, and national life expectancy, all-cause mortality, and cause-specific mortality for 249 causes of death, 1980–2015: a systematic analysis for the Global Burden of Disease Study 2015. The Lancet, 2016 388(10053): p. 1459–1544. 10.1016/S0140-6736(16)31012-1 PMC5388903 27733281

[6] Sheidaei A., et al, National and Subnational Patterns of Cause of Death in Iran 1990–2015: Applied Methods. Arch Iran Med, 2017 20(1): p. 2–11. 28112524

[7] Mohammadi Y., et al, Levels and trends of child and adult mortality rates in the Islamic Republic of Iran, 1990–2013; protocol of the NASBOD study. Arch Iran Med, 2014 17(3): p. 176–81. 24621360

[8] Malta D.C., et al, Mortality due to noncommunicable diseases in Brazil, 1990 to 2015, according to estimates from the Global Burden of Disease study. Sao Paulo Medical Journal, 2017 135(3): p. 213–221. 10.1590/1516-3180.2016.0330050117 28746656 PMC10019842

[9] Santosa A., et al, Achieving a 25% reduction in premature non-communicable disease mortality: the Swedish population as a cohort study. BMC medicine, 2015 13(1): p. 65. 25889300 10.1186/s12916-015-0313-8 PMC4393602

[10] Center M.M., et al, International variation in prostate cancer incidence and mortality rates. European urology, 2012 61(6): p. 1079–1092. 10.1016/j.eururo.2012.02.054 22424666

[11] Oza S., et al, How many deaths are attributable to smoking in the United States? Comparison of methods for estimating smoking-attributable mortality when smoking prevalence changes. Preventive medicine, 2011 52(6): p. 428–433. 21530575 10.1016/j.ypmed.2011.04.007

[12] Torre L.A., et al, Global cancer statistics, 2012. CA: a cancer journal for clinicians, 2015 65(2): p. 87–108. 25651787 10.3322/caac.21262

[13] Cook M.B., et al, Sex disparities in cancer mortality and survival. Cancer Epidemiology and Prevention Biomarkers, 2011 10.3109/1354750X.2011.599042 PMC3153584 21750167

[14] Radkiewicz C., et al, Sex differences in cancer risk and survival: A Swedish cohort study. European Journal of Cancer, 2017 84: p. 130–140. 10.1016/j.ejca.2017.07.013 28802709

[15] Roth G.A., et al, Estimates of global and regional premature cardiovascular mortality in 2025. Circulation, 2015: p. CIRCULATIONAHA. 115.016021. 10.1161/CIRCULATIONAHA.115.016021 26408271

[16] Shiels M.S., et al, Trends in premature mortality in the USA by sex, race, and ethnicity from 1999 to 2014: an analysis of death certificate data. The Lancet, 2017 389(10073): p. 1043–1054. 10.1016/S0140-6736(17)30187-3 PMC5388357 28131493

[17] Ribeiro A.L.P., et al, Cardiovascular health in Brazil: trends and perspectives. Circulation, 2016 133(4): p. 422–433. 10.1161/CIRCULATIONAHA.114.008727 26811272

[18] Macchia A., et al, Premature Cardiovascular death and socioeconomic status in argentina. *On the Opportunities and Challenges of Representing Vulnerable Populations*. Revista Argentina de Cardiología, 2015 83(6).

[19] Zhou M., et al, Cause-specific mortality for 240 causes in China during 1990–2013: a systematic subnational analysis for the Global Burden of Disease Study 2013. The Lancet, 2016 387(10015): p. 251–272. 10.1016/S0140-6736(15)00551-6 26510778

[20] Fang J. and Alderman M.H., Dissociation of hospitalization and mortality trends for myocardial infarction in the United States from 1988 to 1997. The American journal of medicine, 2002 113(3): p. 208–214. 12208379 10.1016/s0002-9343(02)01172-5

[21] Makdisse M.R.P., et al, Pharmacological therapy for myocardial infarction in the elderly: an 8-year analysis. Arquivos brasileiros de cardiologia, 2002 78(4): p. 369–373. 10.1590/s0066-782x2002000400003 12011952

[22] Ishihara M., et al, Fifteen-year trend in the treatment and outcome of acute myocardial infarction in Japan. Circulation journal, 2002 66(2): p. 178–181. 11999644 10.1253/circj.66.178

[23] Radišauskas R., et al, Morbidity and mortality from the major cardiovascular diseases in Kaunas population from 1983 to 2002. Medicina (Kaunas), 2003 39(12): p. 1208–14. 14704510

[24] Abildstrom S., et al, Trends in incidence and case fatality rates of acute myocardial infarction in Denmark and Sweden. Heart, 2003 89(5): p. 507–511. 12695453 10.1136/heart.89.5.507 PMC1767620

[25] Varmaghani M., et al, Death Specific Rate Due to Asthma and Chronic Obstructive Pulmonary Disease in Iran. The clinical respiratory journal, 2018. 10.1111/crj.12776 29405628

[26] Buist A.S., et al, International variation in the prevalence of COPD (the BOLD Study): a population-based prevalence study. The Lancet, 2007 370(9589): p. 741–750. 10.1016/S0140-6736(07)61377-4 17765523

[27] De Marco R., et al, The coexistence of asthma and chronic obstructive pulmonary disease (COPD): prevalence and risk factors in young, middle-aged and elderly people from the general population. PloS one, 2013 8(5): p. e62985 10.1371/journal.pone.0062985 23675448 PMC3651288

[28] Atash F., The deterioration of urban environments in developing countries: Mitigating the air pollution crisis in Tehran, Iran. Cities, 2007 24(6): p. 399–409.

[29] Ko F.W., et al, Temporal relationship between air pollutants and hospital admissions for chronic obstructive pulmonary disease in Hong Kong. Thorax, 2007 62(9): p. 780–785. 10.1136/thx.2006.076166 17311838 PMC2117326

[30] Lee S., Wong W., and Lau Y., Association between air pollution and asthma admission among children in Hong Kong. Clinical & Experimental Allergy, 2006 36(9): p. 1138–1146. 16961713 10.1111/j.1365-2222.2006.02555.x PMC1618810

[31] Malhão T.A., et al, Sex differences in diabetes mellitus mortality trends in Brazil, 1980–2012. PloS one, 2016 11(6): p. e0155996 10.1371/journal.pone.0155996 27275600 PMC4898826

[32] Roche M.M. and Wang P.P., Sex differences in all-cause and cardiovascular mortality, hospitalization for individuals with and without diabetes, and patients with diabetes diagnosed early and late. Diabetes Care, 2013: p. DC_x121272. 10.2337/dc12-1272 PMC3747934 23564923

[33] Xi B., et al, Secular trends in the prevalence of general and abdominal obesity among Chinese adults, 1993–2009. Obesity reviews, 2012 13(3): p. 287–296. 10.1111/j.1467-789X.2011.00944.x 22034908 PMC3276709

[34] Li Y., et al, Risk factors for noncommunicable chronic diseases in women in China: surveillance efforts. Bulletin of the World Health Organization, 2013 91: p. 650–660. 10.2471/BLT.13.117549 24101781 PMC3790222

[35] Zhai F., et al, Prospective study on nutrition transition in China. Nutrition reviews, 2009 67(suppl_1): p. S56–S61. 19453679 10.1111/j.1753-4887.2009.00160.x

[36] Barreto S.M., et al, The increase of diabetes mortality burden among Brazilian adults. Revista Panamericana de Salud Pública, 2007 22(4): p. 239–245. 18078585 10.1590/s1020-49892007000900003

[37] Malta D.C., et al, Trends in Self-reported Diabetes among adults in Brazilian state capitals, 2006–2012. Epidemiologia e Serviços de Saúde, 2014 23(4): p. 753–760.

[38] Khosravi Shadmani F., *National and subnational levels achievement to Sustainable Development Goals(SDGs) for non-communicable diseases in Iran: modeling study with scenario-based projection*. 2018, Kerman University of Medical Sciences.

[39] Jackson E.A., et al, Changes over time in the use of aspirin in patients hospitalized with acute myocardial infarction (1975 to 1997): a population-based perspective. American heart journal, 2002 144(2): p. 259–268. 12177643 10.1067/mhj.2002.123837

