# Supplementary material for: Trend and projection of mortality rate due to non-communicable diseases in Iran: A modeling study
Source: PLoS One. 2019 Feb 14;14(2):e0211622. doi: 10.1371/journal.pone.0211622 (PMC6375574; doi:10.1371/journal.pone.0211622)
Supplement: S1 Table — (DOCX) [file pone.0211622.s001.docx]

|  | Cancers | Cardiovascular diseases | Ashtma & COPD | Diabetes |
| --- | --- | --- | --- | --- |
| **Provinces** |  |  |  |  |
| Iran | -43.4 | -41.9 | -10.9 | -4.3 |
| Markazi | -39.3 | -40.5 | -5.1 | 9.3 |
| Gilan | -42.5 | -41.2 | -10.4 | -2.5 |
| Mazandaran | -44.5 | -42 | -12.8 | -8.7 |
| East Azerbaijan | -46.4 | -43.2 | -14.7 | -14.1 |
| West Azerbaijan | -50.2 | -45.3 | -19.4 | -25.2 |
| Kermanshah | -44.7 | -42.7 | -12.2 | -8.7 |
| Khuzestan | -40.4 | -40.5 | -6.9 | 5.1 |
| Fars | -39.4 | -40.1 | -5.6 | 8.6 |
| Kerman | -46.7 | -43.3 | -14.9 | -14.9 |
| Razavi Khorasan | -43.2 | -42.1 | -10.1 | -3.6 |
| Isfahan | -42.3 | -42.3 | -8.0 | -0.0 |
| Sistan and Baluchestan | -48.3 | -44.6 | -16.5 | -19.5 |
| Kurdistan | -52.5 | -46.3 | -22.6 | -31.8 |
| Hamadan | -41.6 | -40.9 | -8.8 | 0.8 |
| Chaharmahal and Bakhtiari | -42.6 | -41.4 | -9.8 | -2.2 |
| Lorestan | -50.3 | -45.1 | -19.8 | -25.7 |
| Ilam | -42.1 | -41.0 | -9.6 | -0.9 |
| Kohgiluyeh and Boyer-Ahmad | -47.6 | -43.8 | -16.6 | -1.0 |
| Bushehr | -32.9 | -37.3 | 2.3 | 31.8 |
| Zanjan | -58.2 | -49.0 | -30.6 | -46.8 |
| Semnan | -37.0 | -38.6 | -3.4 | 16.2 |
| Yazd | -39.9 | -39.7 | -7.2 | 6.0 |
| Hormozgan | -31.5 | -36.6 | 3.8 | 36.9 |
| Tehran | -41.8 | -41.9 | -7.4 | 1.5 |
| Ardabil | -46.9 | -43.2 | -15.8 | -16 |
| Qom | -43.2 | -42.2 | -9.8 | -3.3 |
| Qazvin | -42.0 | -42.0 | -7.9 | 0.7 |
| Golestan | -37.5 | -38.9 | -3.9 | 14.5 |
| North Khorasan | -46.8 | -43.1 | -15.7 | -15.9 |
| South Khorasan | -42.6 | -40.8 | -10.8 | -2.9 |
| Alborz | -39.4 | -40.2 | -5.5 | 8.7 |

**S1 Table1.** percent of change for NCDs in both sexes for provinces in Iran, by cause, from 2001 to 2015
