# Supplementary figures and images for: Trend and projection of mortality rate due to non-communicable diseases in Iran: A modeling study
Source: PLoS One. 2019 Feb 14;14(2):e0211622. doi: 10.1371/journal.pone.0211622 (PMC6375574; doi:10.1371/journal.pone.0211622)

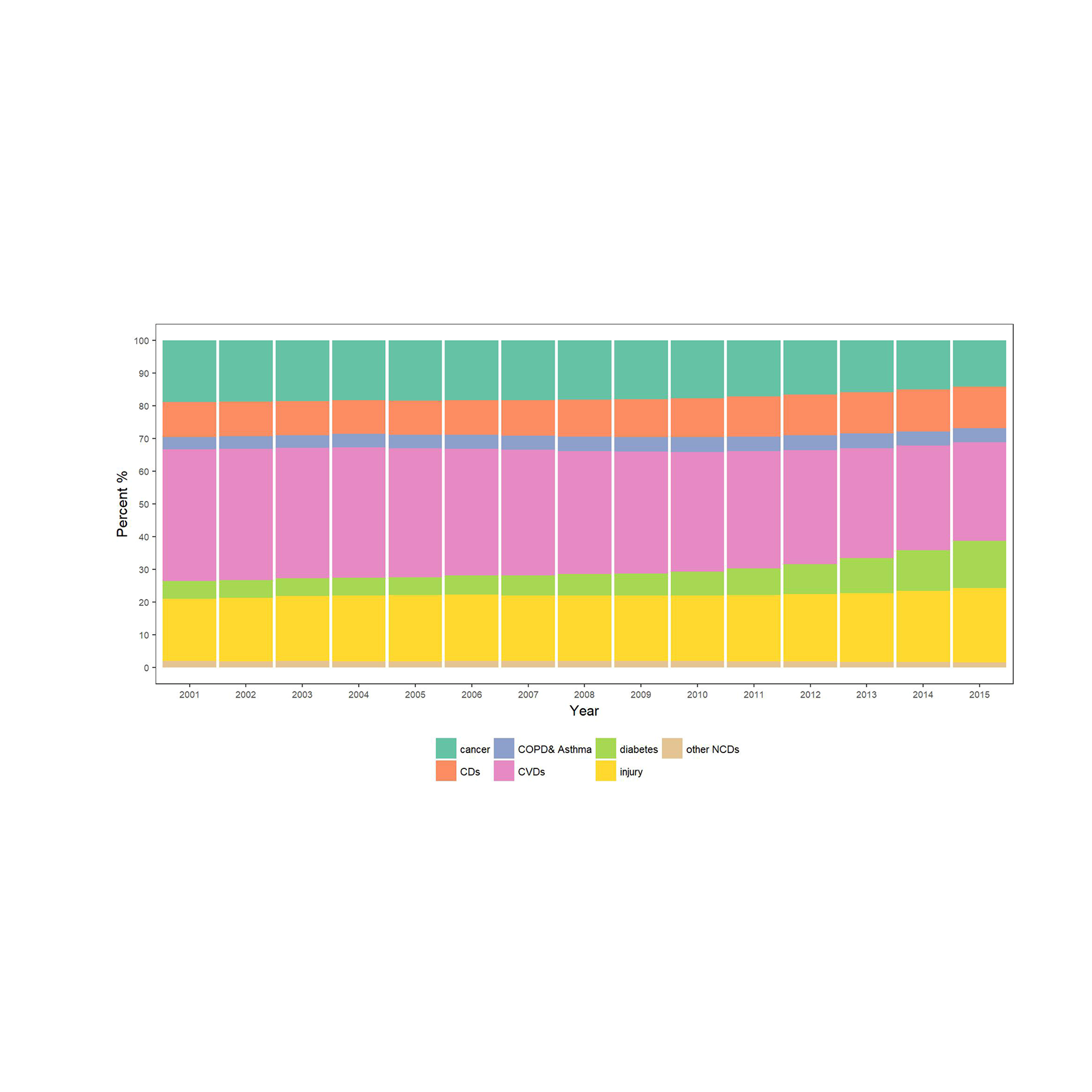

Supplement: S1 Fig — (TIF) [file pone.0211622.s002.tif]

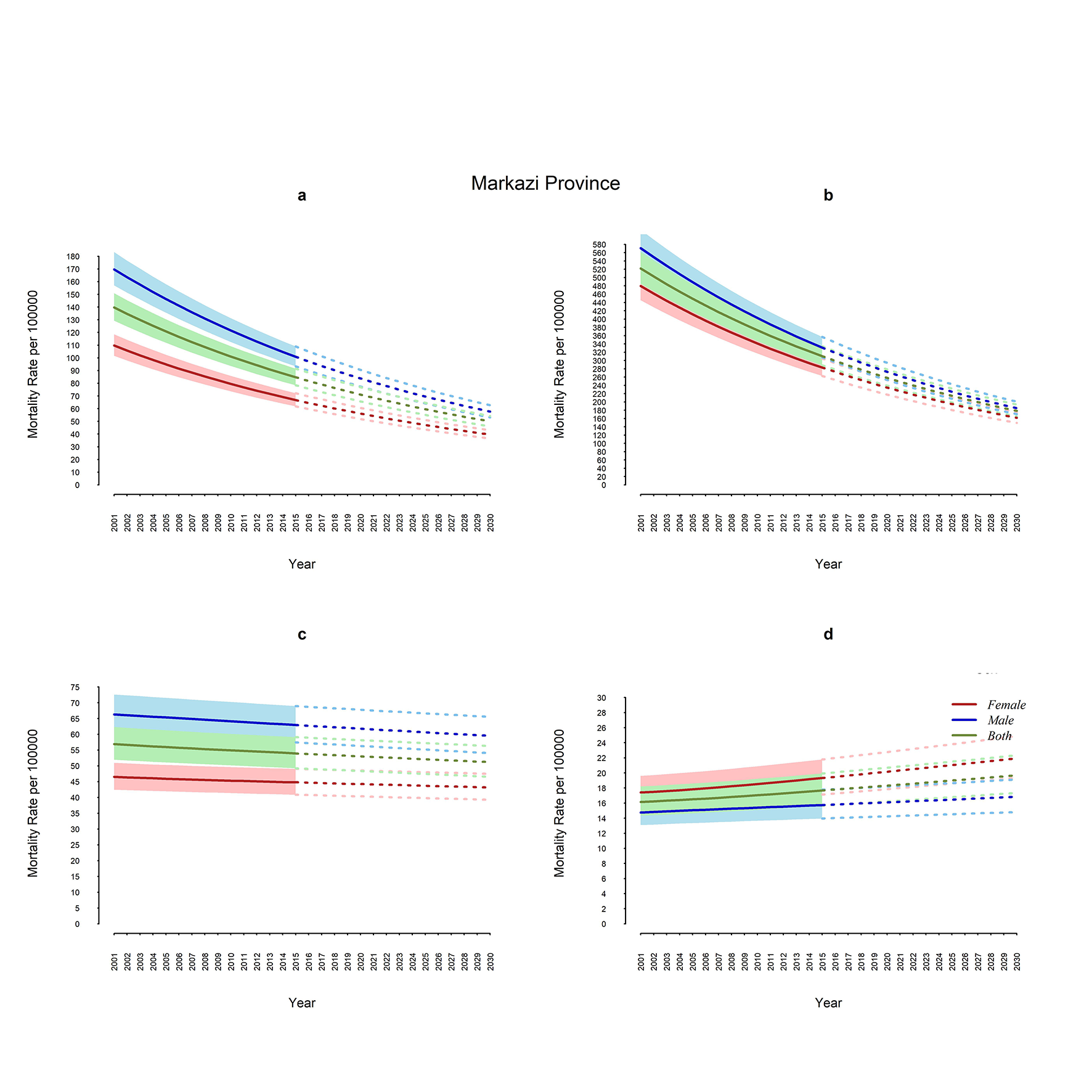

Supplement: S2 Fig — a) Cancer, b) CVDs, c) Asthma and COPD, d) Diabetes.Markazi province. (TIF) [file pone.0211622.s003.tif]

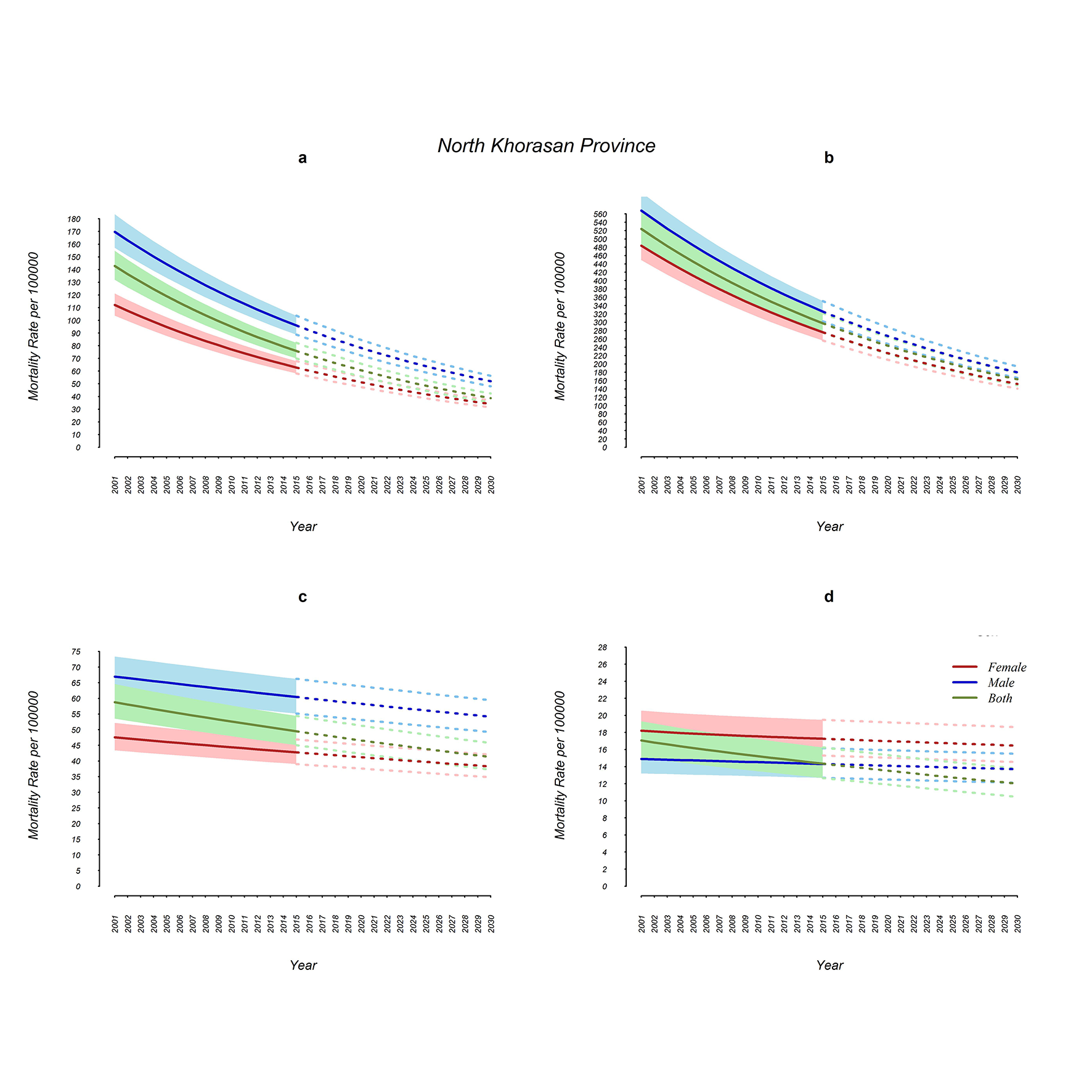

Supplement: S3 Fig — a) Cancer, b) CVDs, c) Asthma and COPD, d) Diabetes. North Khorasan province. (TIF) [file pone.0211622.s004.tif]

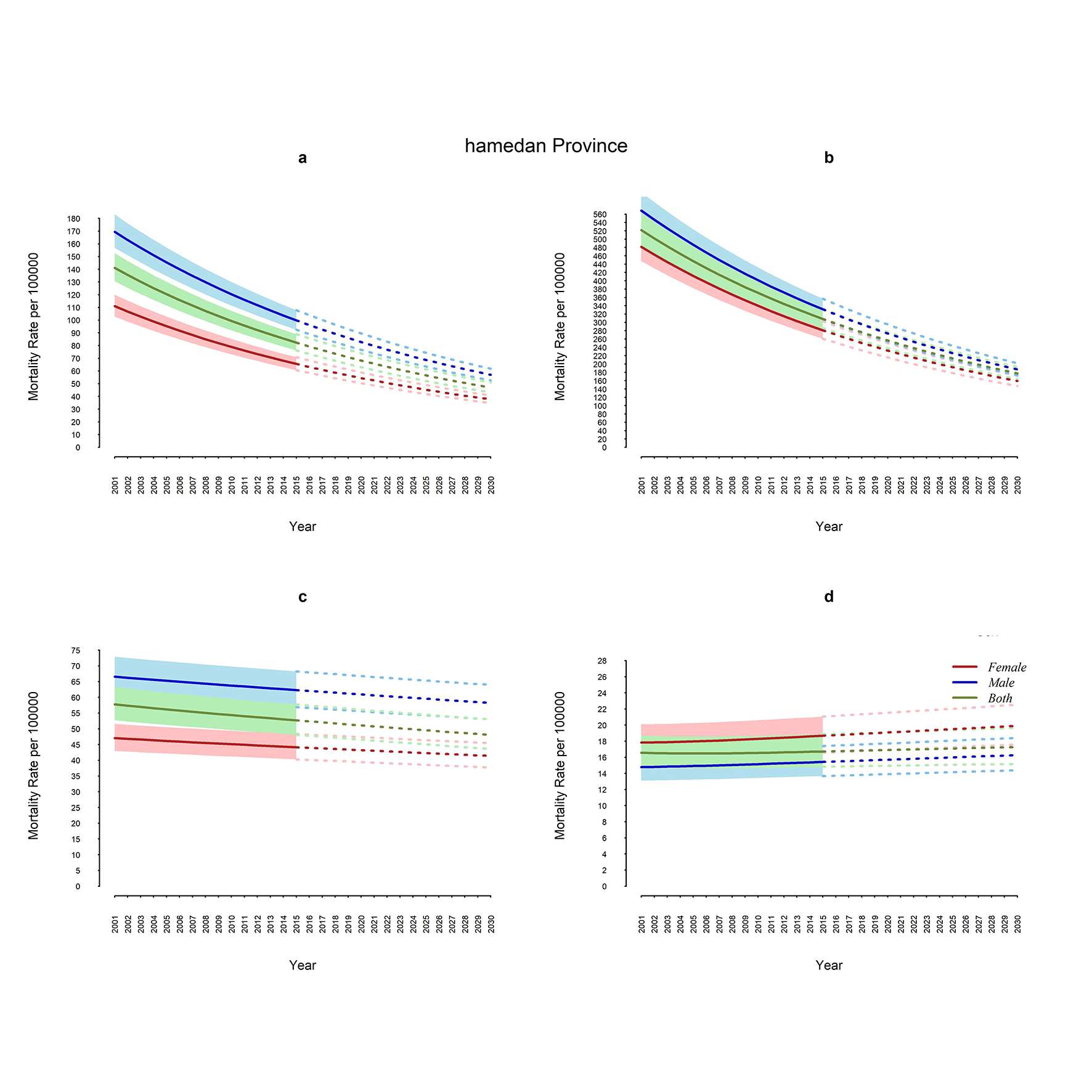

Supplement: S4 Fig — a) Cancer, b) CVDs, c) Asthma and COPD, d) Diabetes. Hamedan province. (TIF) [file pone.0211622.s005.tif]

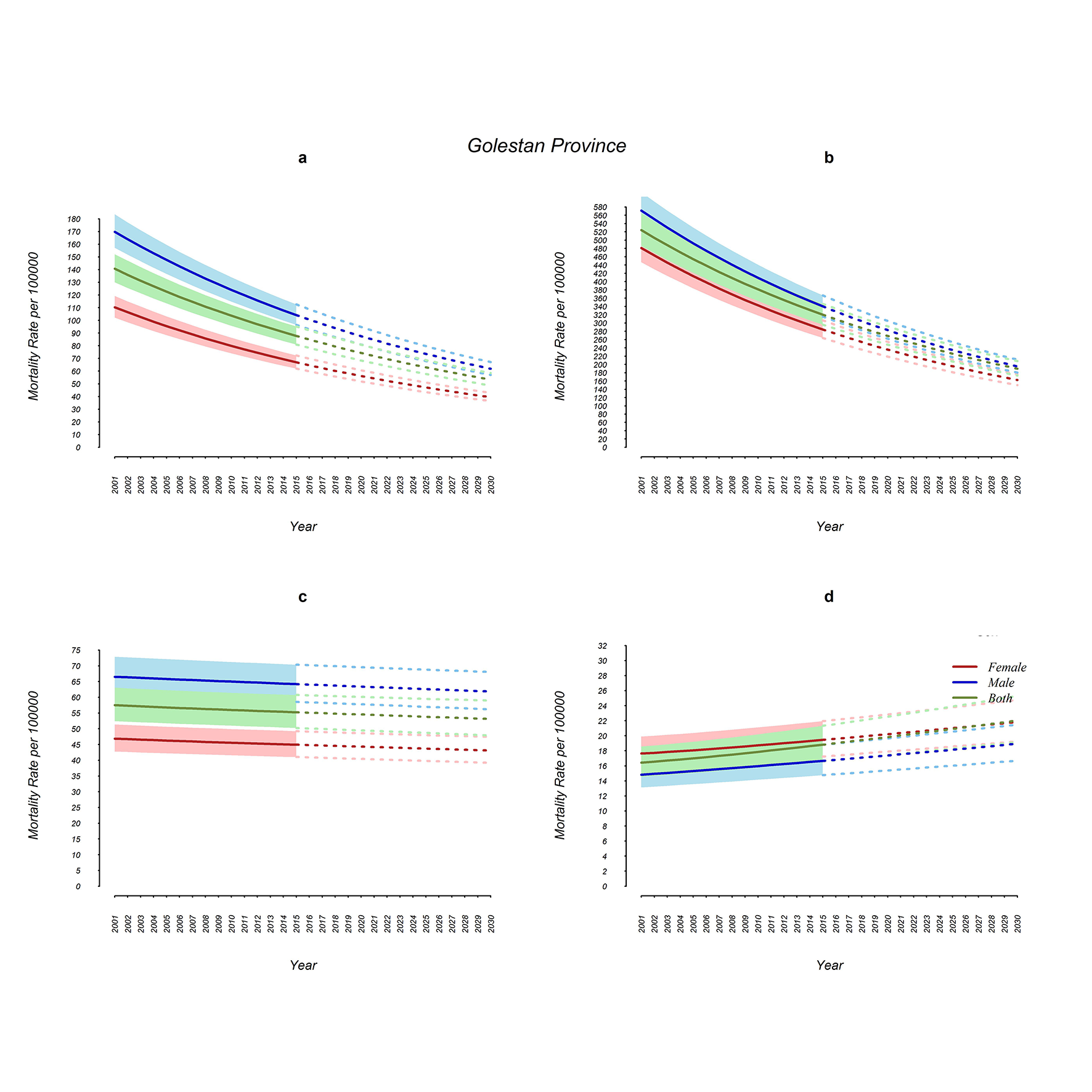

Supplement: S5 Fig — a) Cancer, b) CVDs, c) Asthma and COPD, d) Diabetes. Golestan province. (TIF) [file pone.0211622.s006.tif]

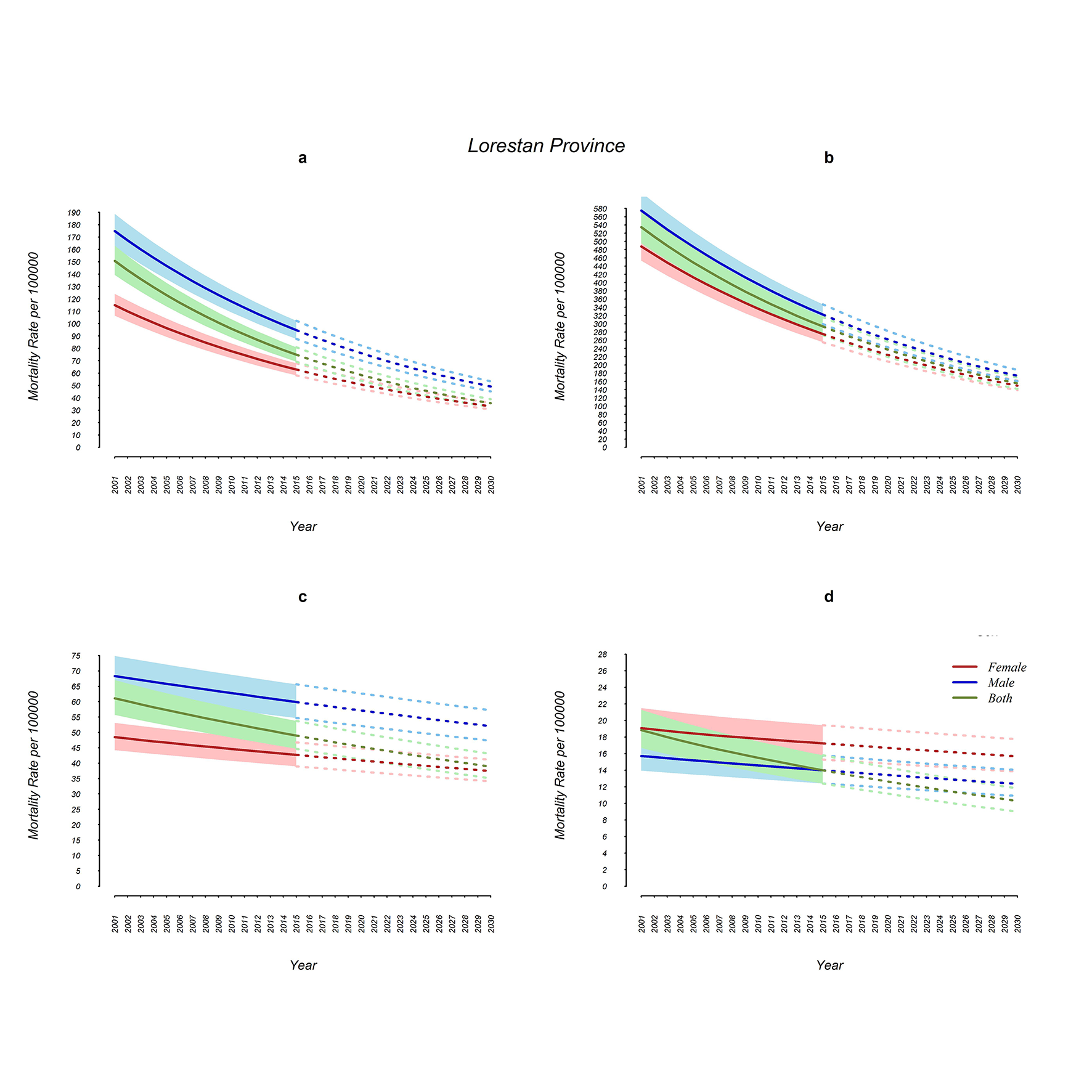

Supplement: S6 Fig — a) Cancer, b) CVDs, c) Asthma and COPD, d) Diabetes. Lorestan province. (TIF) [file pone.0211622.s007.tif]

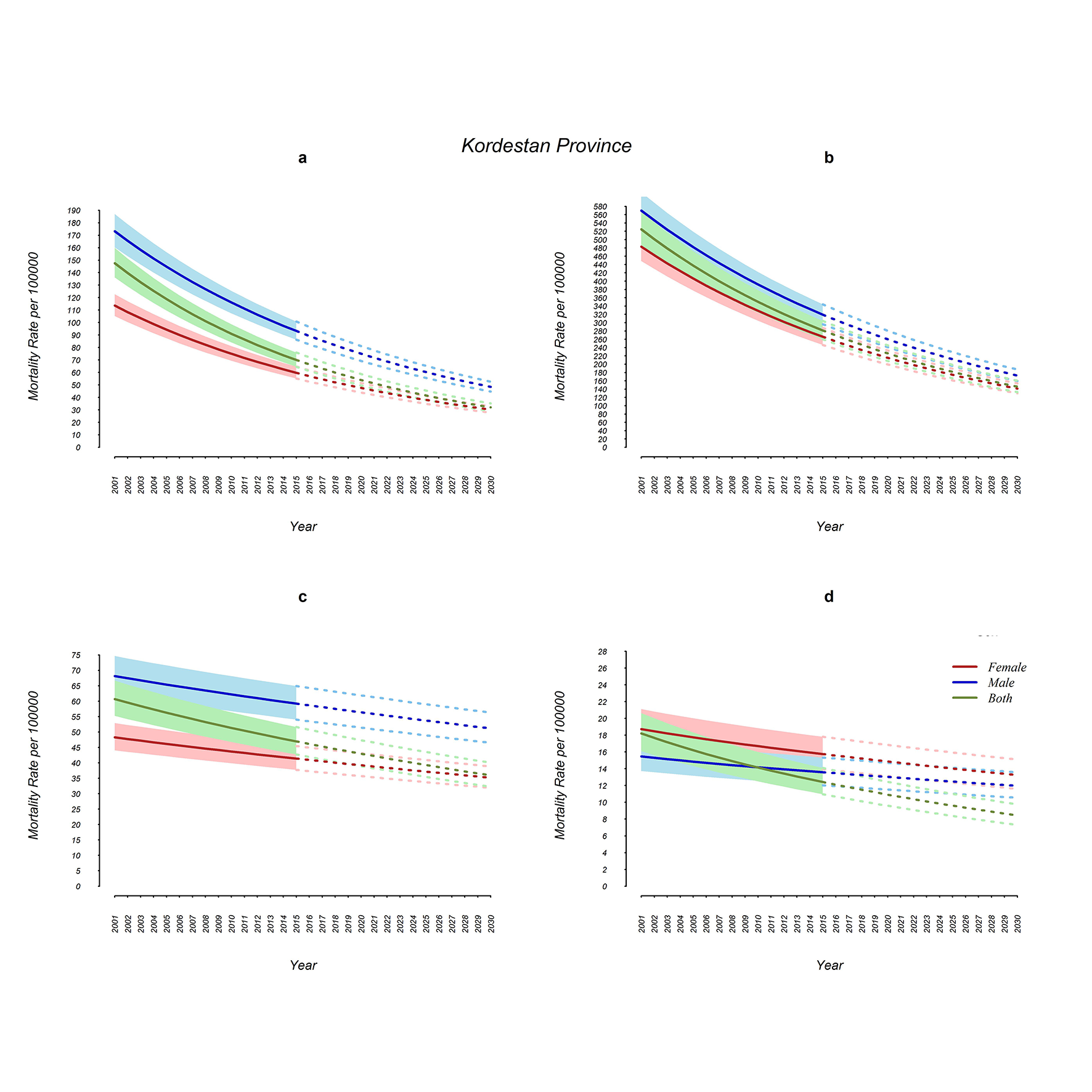

Supplement: S7 Fig — a) Cancer, b) CVDs, c) Asthma and COPD, d) Diabetes. Kordestan province. (TIF) [file pone.0211622.s008.tif]

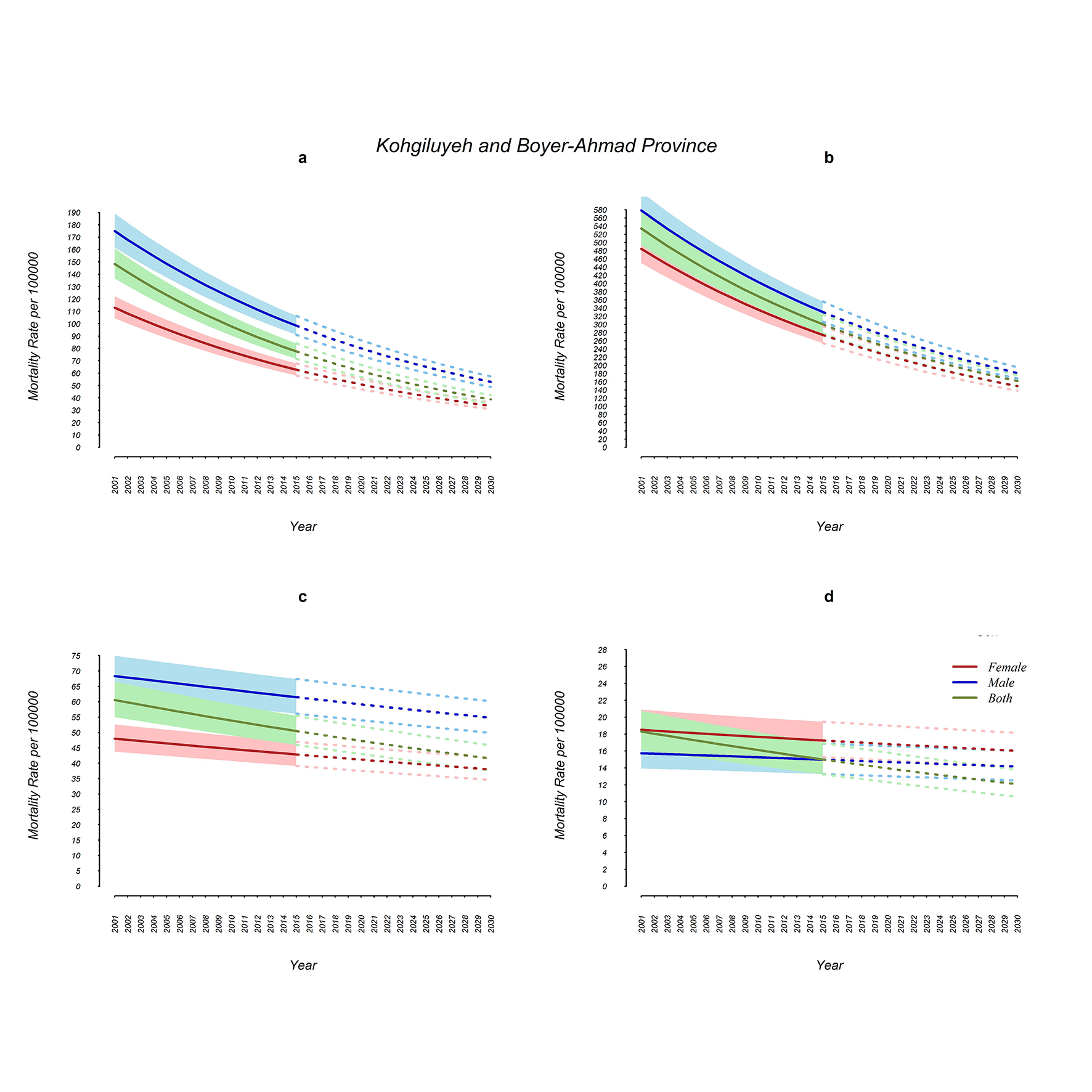

Supplement: S8 Fig — a) Cancer, b) CVDs, c) Asthma and COPD, d) Diabetes. Kohgiluyeh and Boyer- Ahmad province. (TIF) [file pone.0211622.s009.tif]

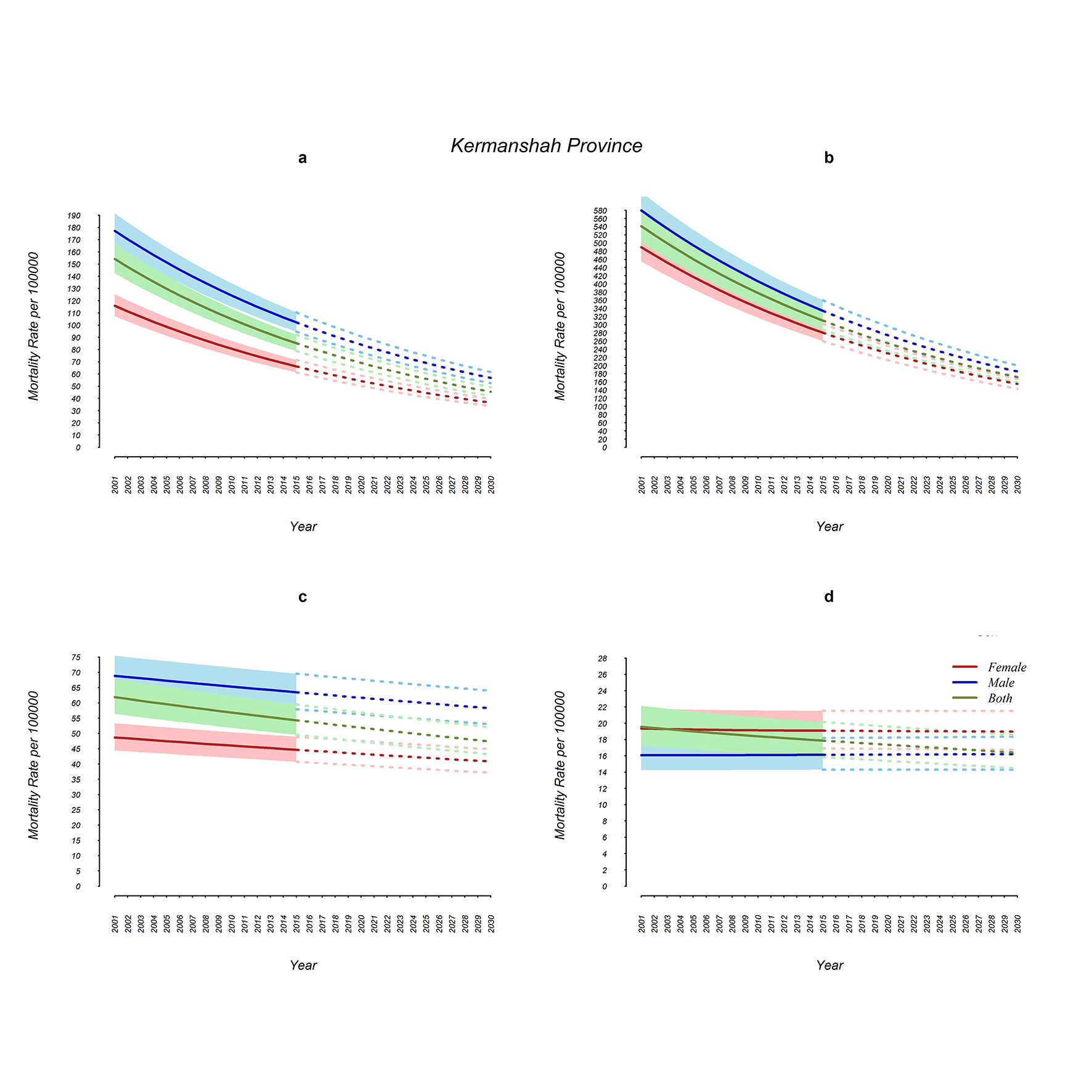

Supplement: S9 Fig — a) Cancer, b) CVDs, c) Asthma and COPD, d) Diabetes. Kermanshah province. (TIF) [file pone.0211622.s010.tif]

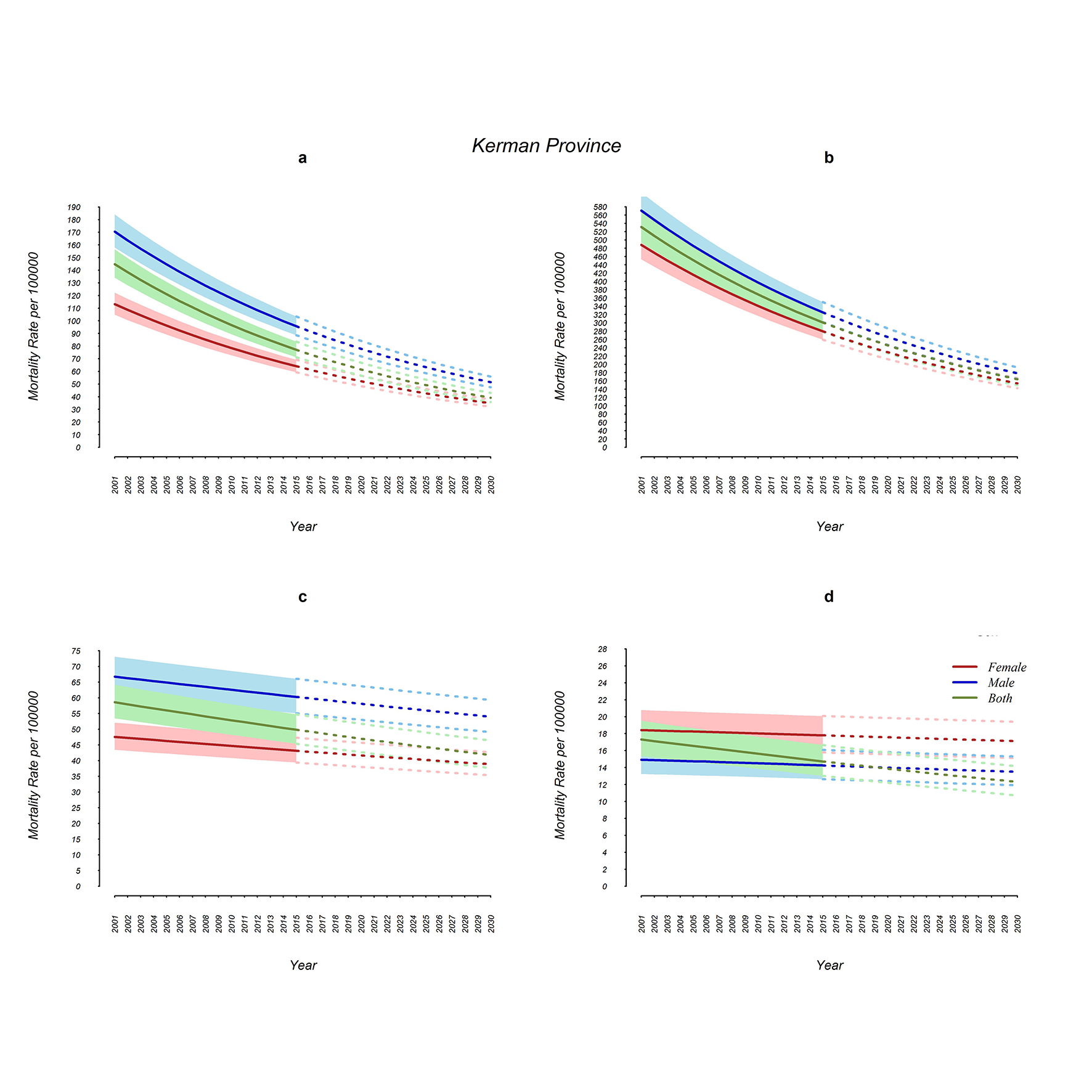

Supplement: S10 Fig — a) Cancer, b) CVDs, c) Asthma and COPD, d) Diabetes. Kerman province. (TIF) [file pone.0211622.s011.tif]

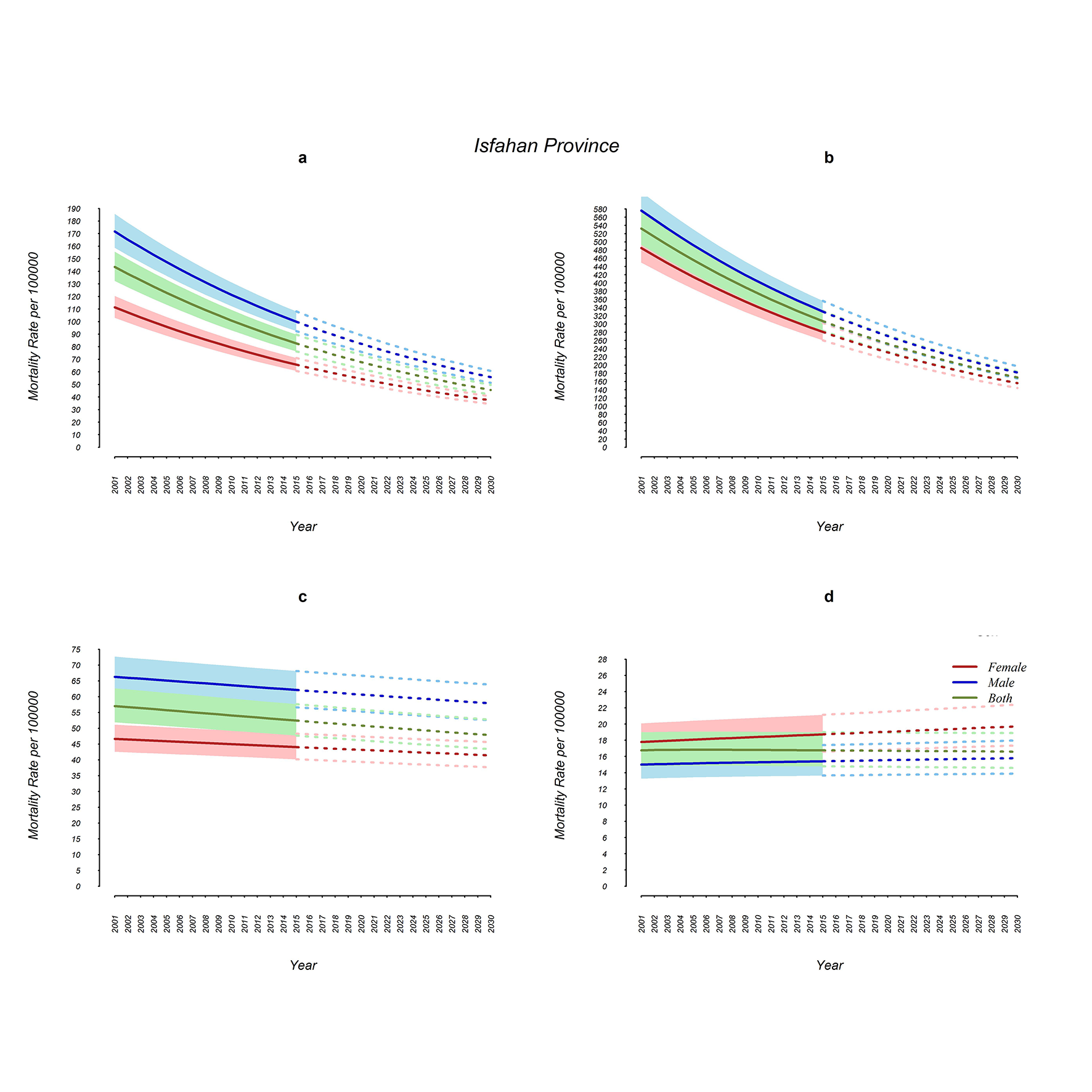

Supplement: S11 Fig — a) Cancer, b) CVDs, c) Asthma and COPD, d) Diabetes. Isfahan province. (TIF) [file pone.0211622.s012.tif]

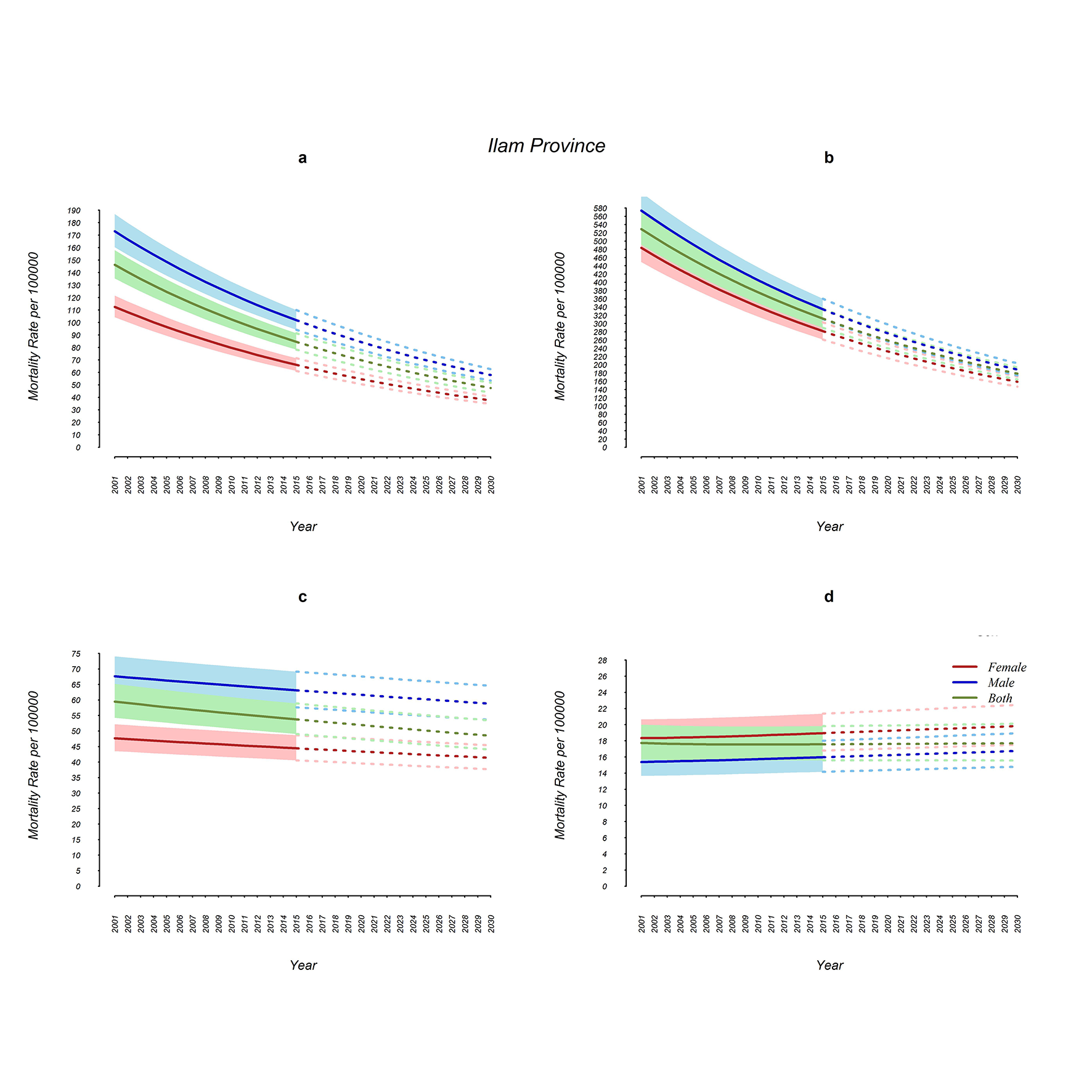

Supplement: S12 Fig — a) Cancer, b) CVDs, c) Asthma and COPD, d) Diabetes. Ilam province. (TIF) [file pone.0211622.s013.tif]

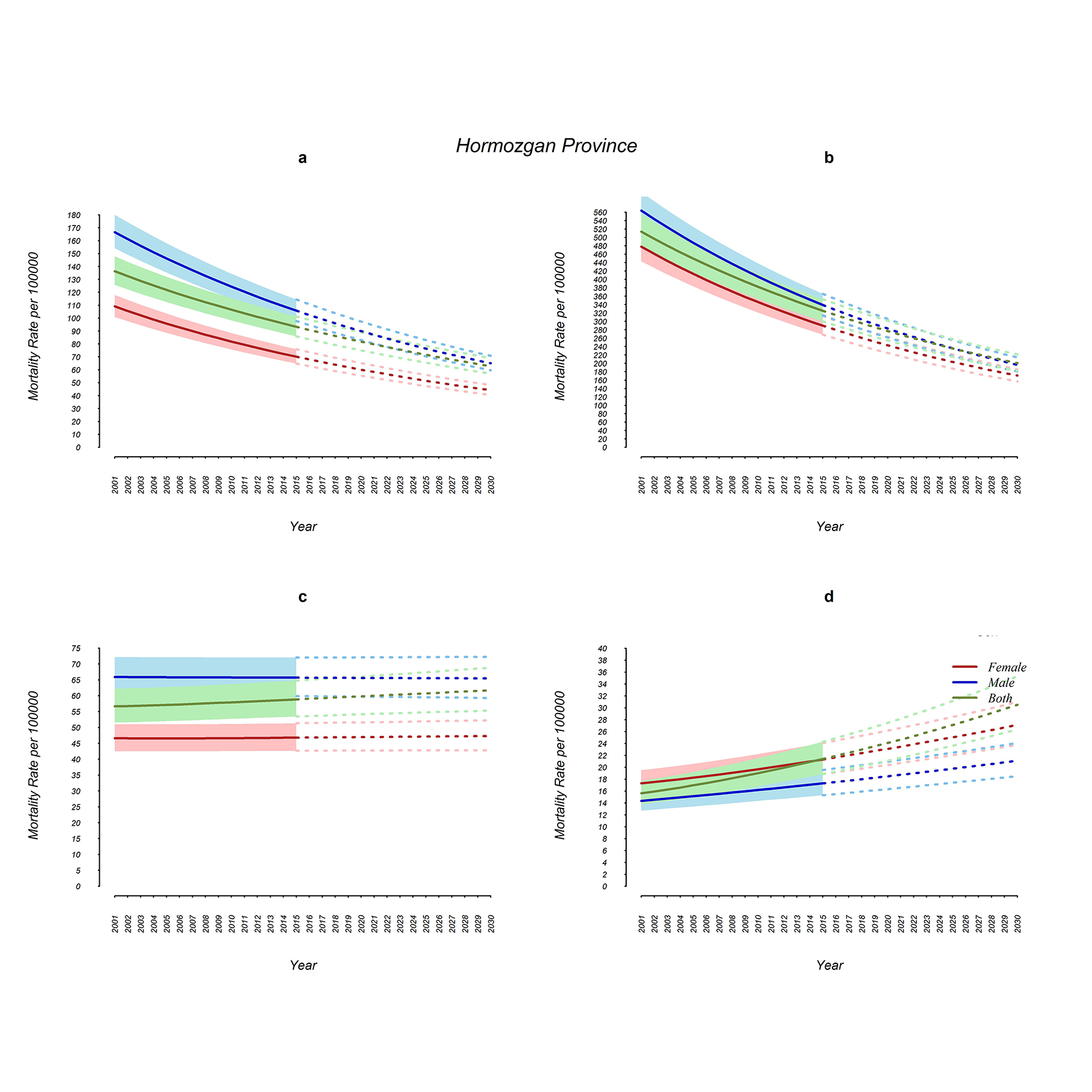

Supplement: S13 Fig — a) Cancer, b) CVDs, c) Asthma and COPD, d) Diabetes. Hormozgan province. (TIF) [file pone.0211622.s014.tif]

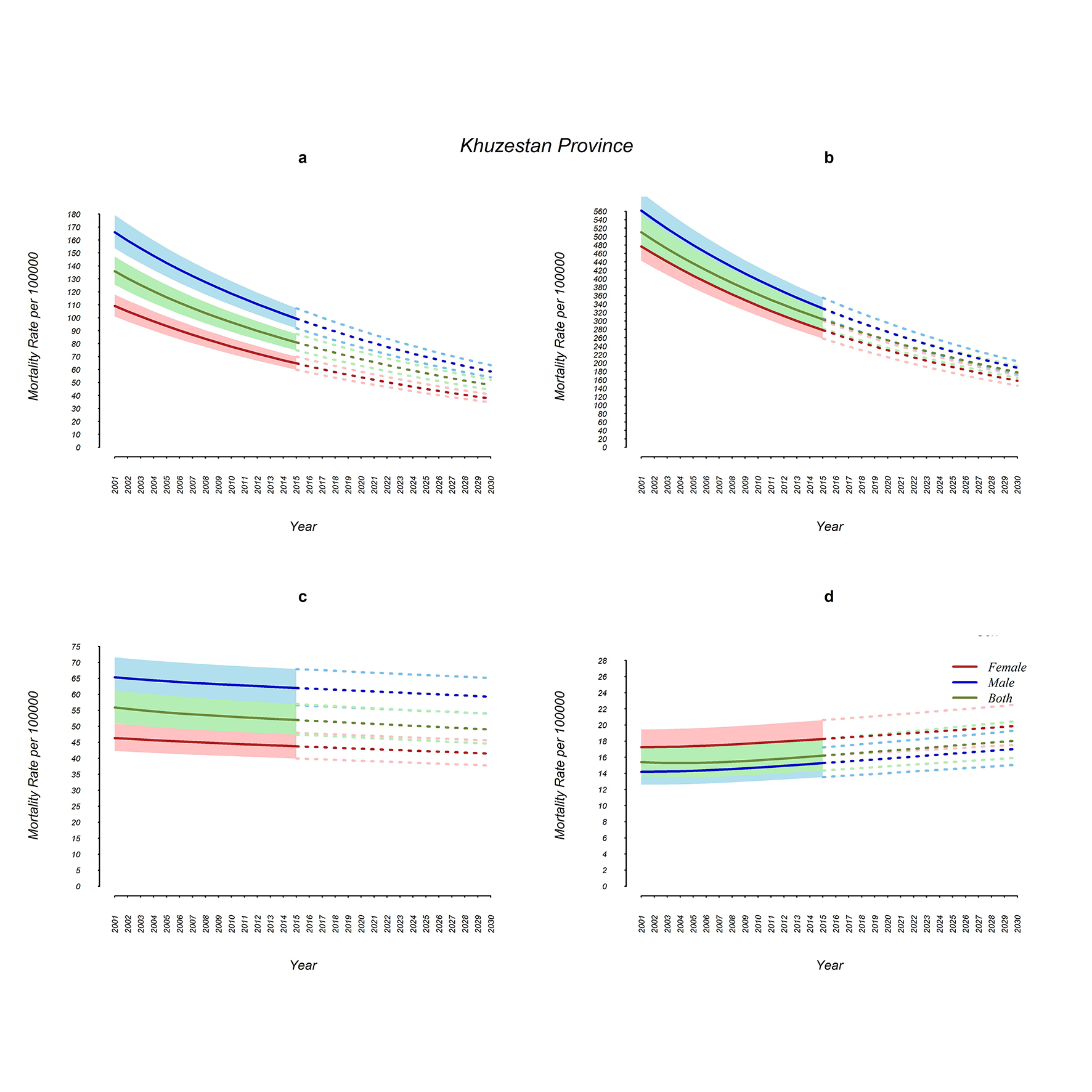

Supplement: S14 Fig — a) Cancer, b) CVDs, c) Asthma and COPD, d) Diabetes. Khuzestan province. (TIF) [file pone.0211622.s015.tif]

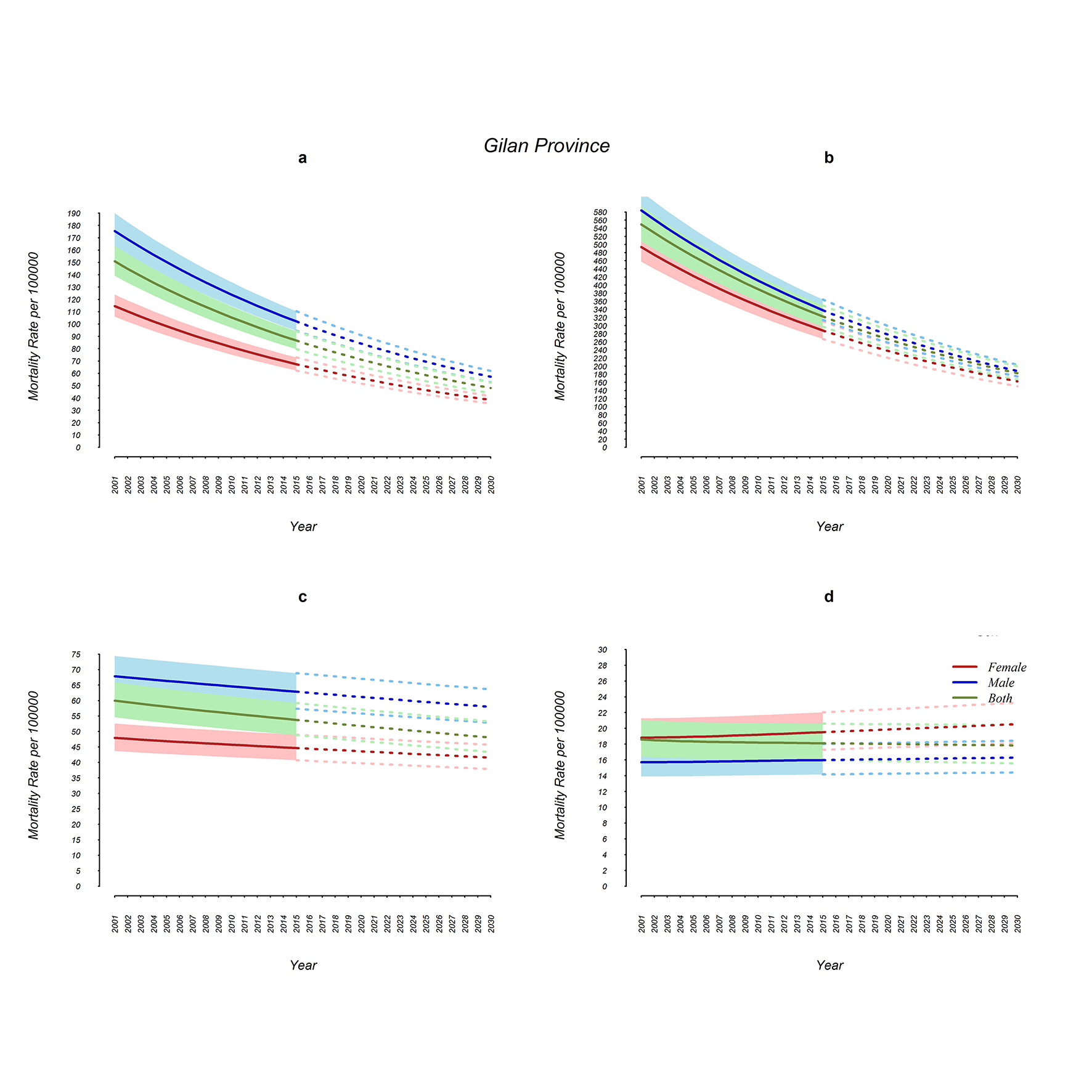

Supplement: S15 Fig — a) Cancer, b) CVDs, c) Asthma and COPD, d) Diabetes. Gilan province. (TIF) [file pone.0211622.s016.tif]

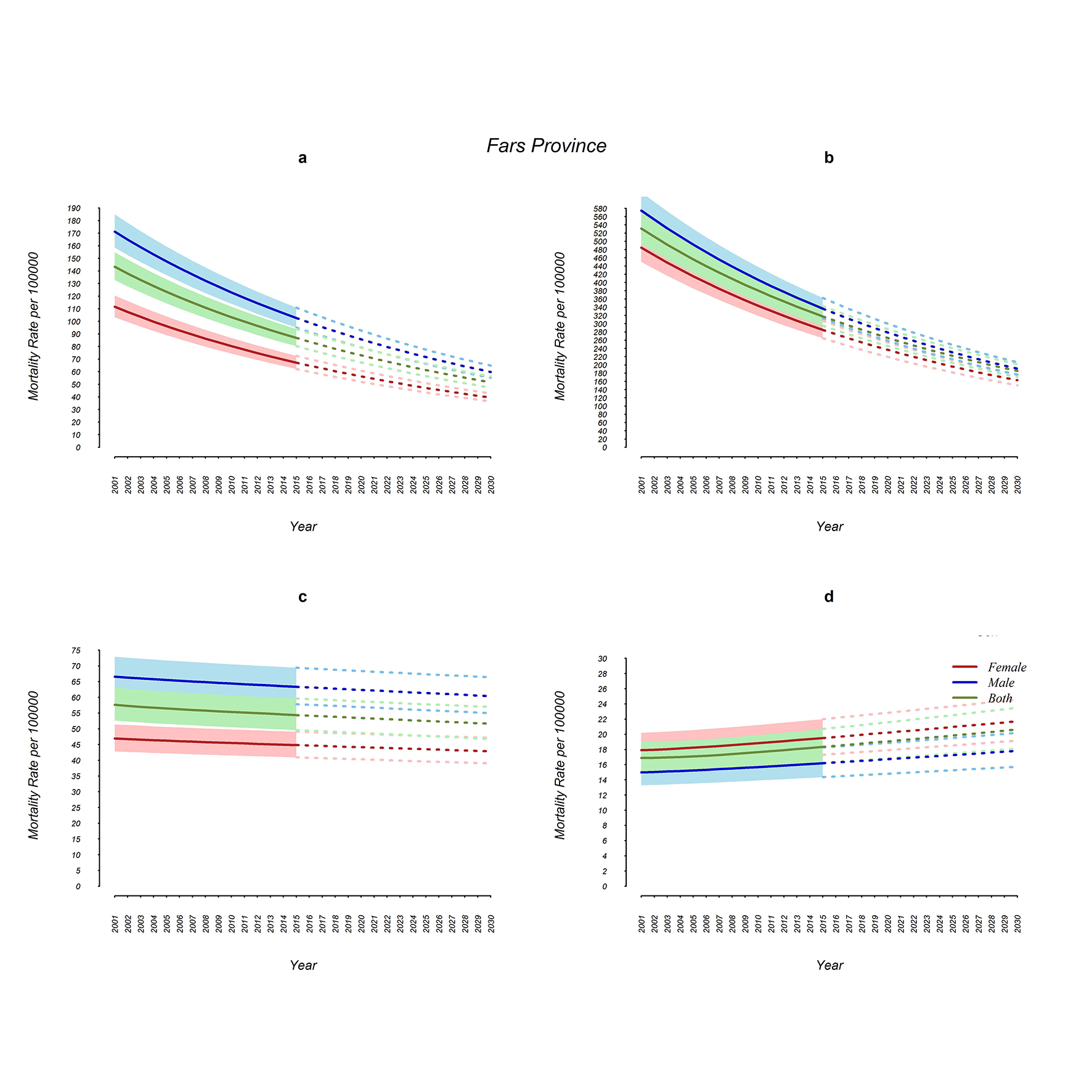

Supplement: S16 Fig — a) Cancer, b) CVDs, c) Asthma and COPD, d) Diabetes. Fars province. (TIF) [file pone.0211622.s017.tif]

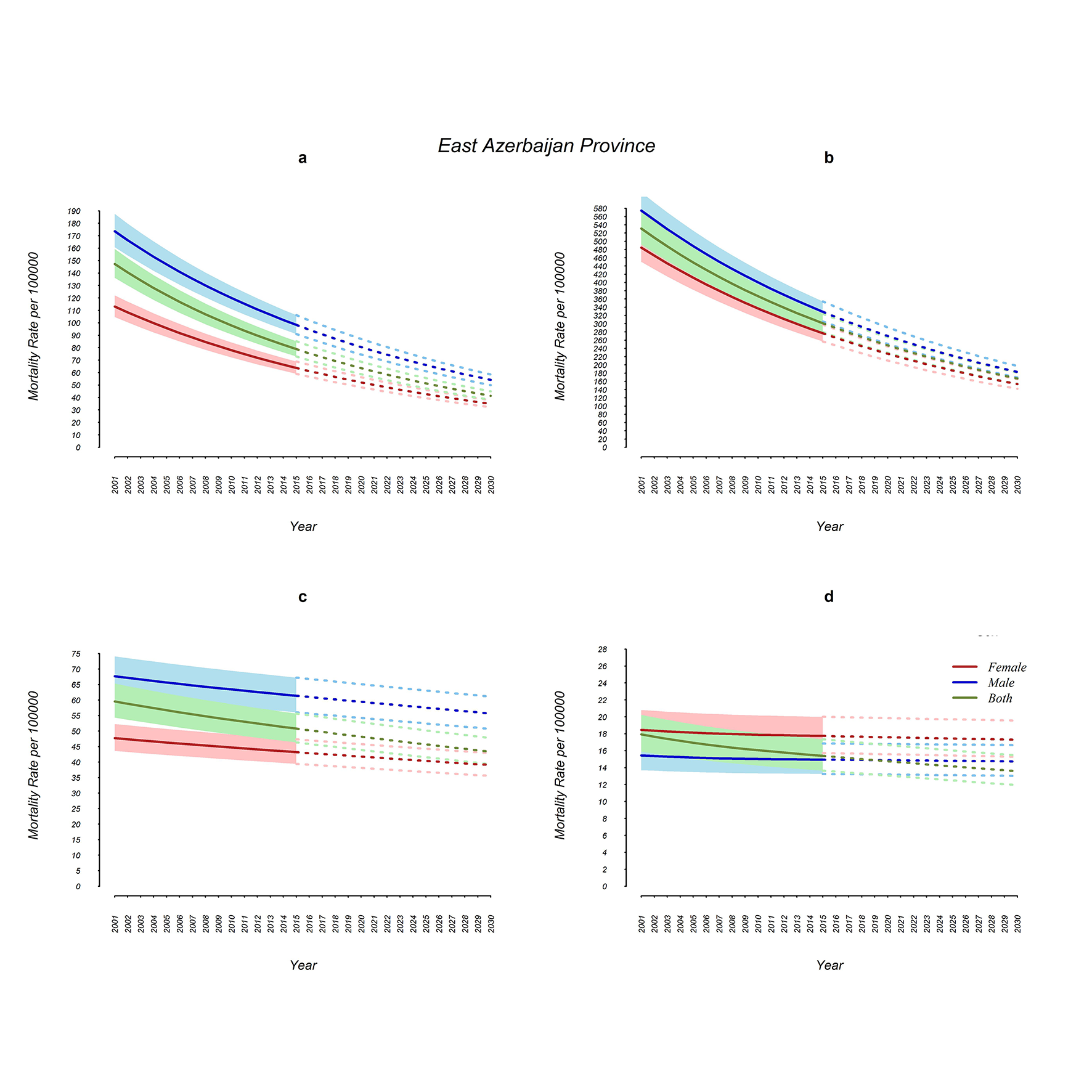

Supplement: S17 Fig — a) Cancer, b) CVDs, c) Asthma and COPD, d) Diabetes. East Azarbaijan province. (TIF) [file pone.0211622.s018.tif]

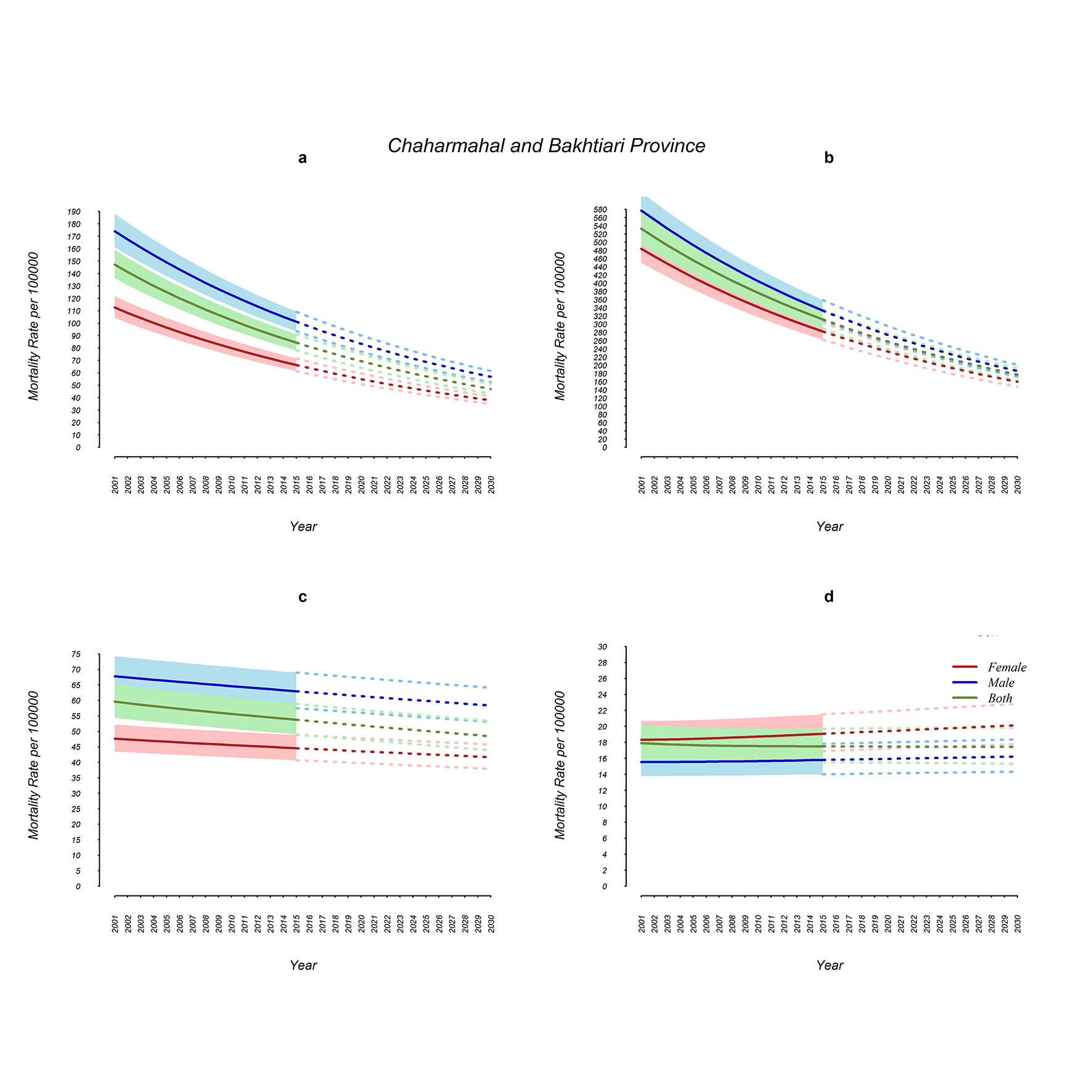

Supplement: S18 Fig — a) Cancer, b) CVDs, c) Asthma and COPD, d) Diabetes. Chaharmahal and Bakhtiari province. (TIF) [file pone.0211622.s019.tif]

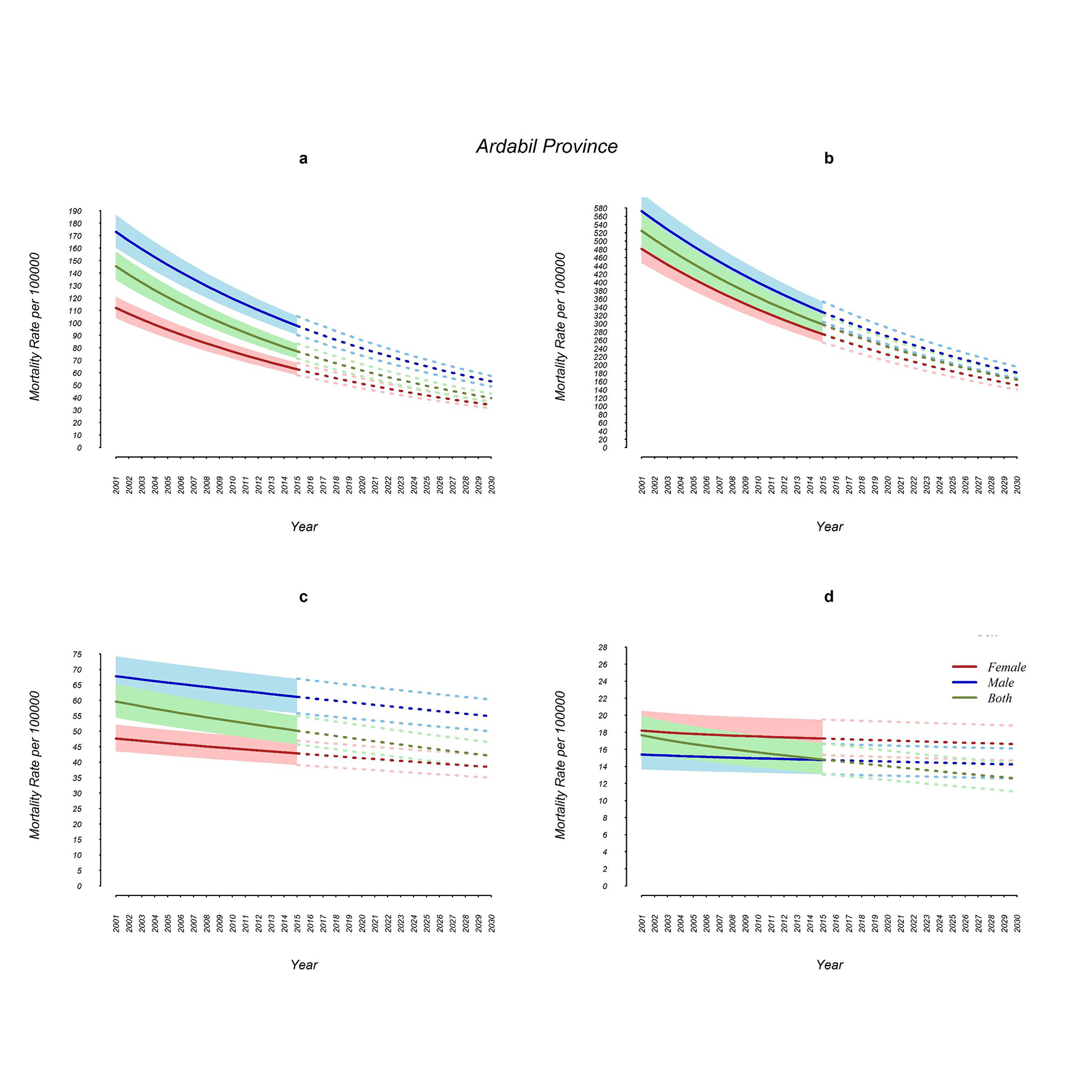

Supplement: S19 Fig — a) Cancer, b) CVDs, c) Asthma and COPD, d) Diabetes. Ardebil province. (TIF) [file pone.0211622.s020.tif]

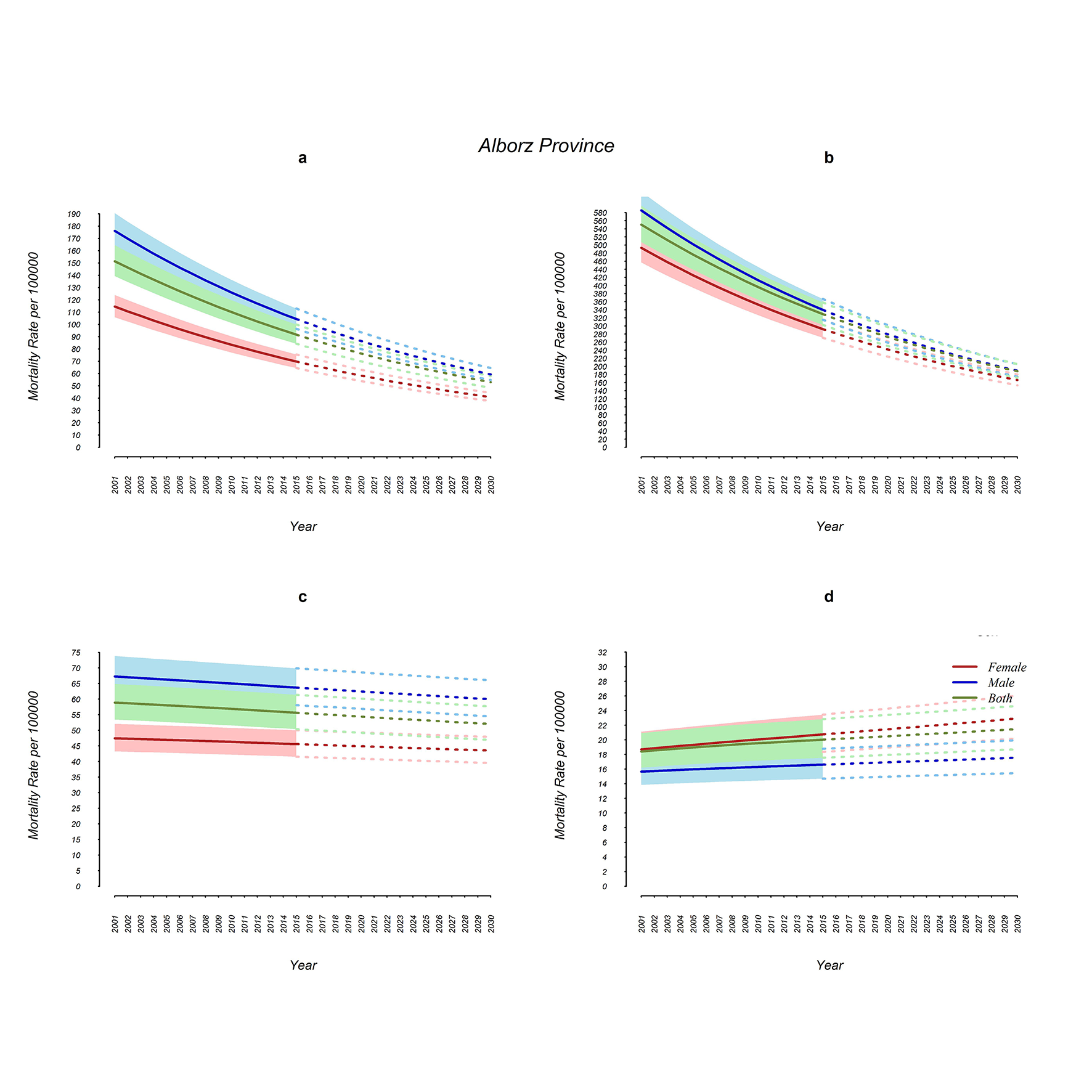

Supplement: S20 Fig — a) Cancer, b) CVDs, c) Asthma and COPD, d) Diabetes. Alborz province. (TIF) [file pone.0211622.s021.tif]

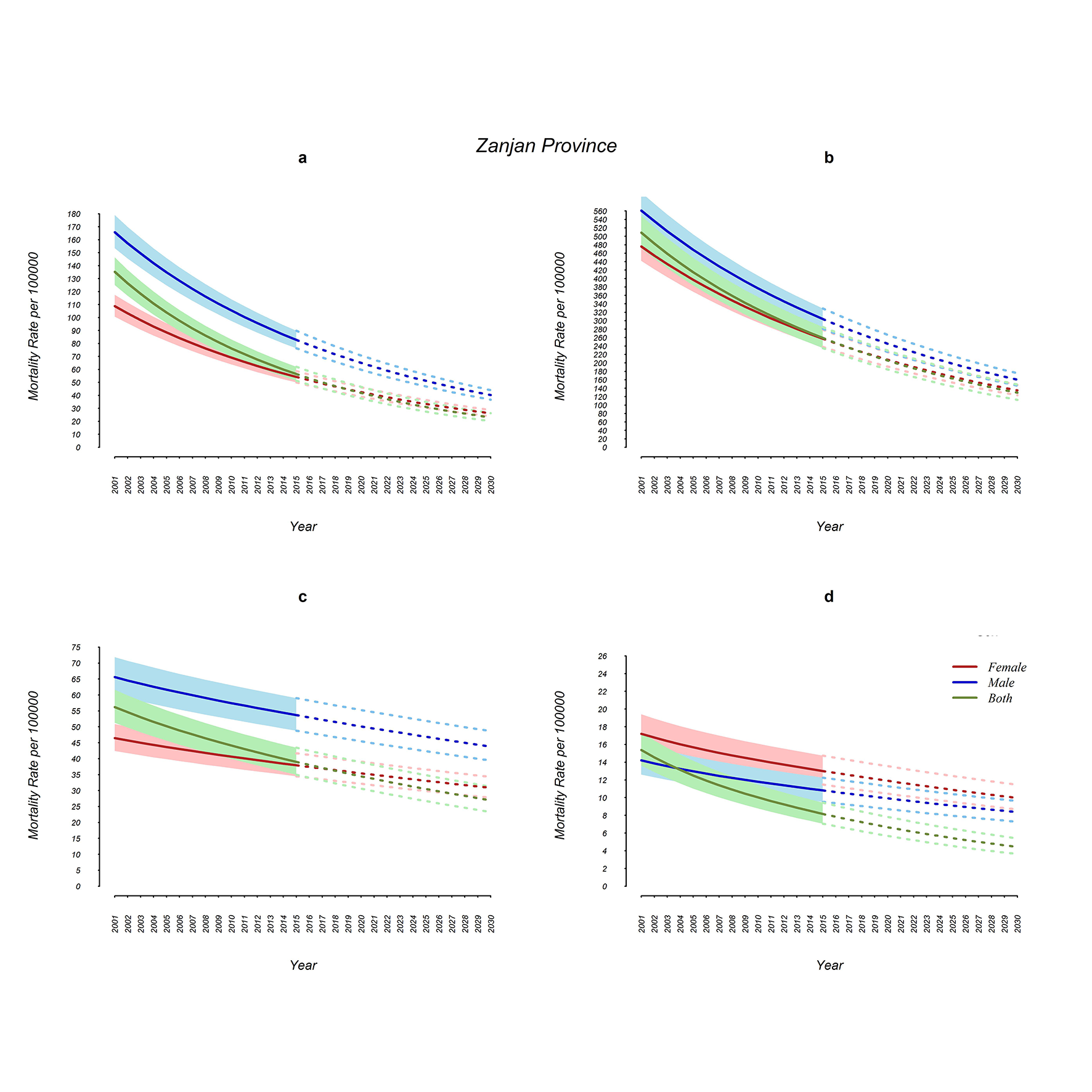

Supplement: S21 Fig — a) Cancer, b) CVDs, c) Asthma and COPD, d) Diabetes. Zanjan province. (TIF) [file pone.0211622.s022.tif]

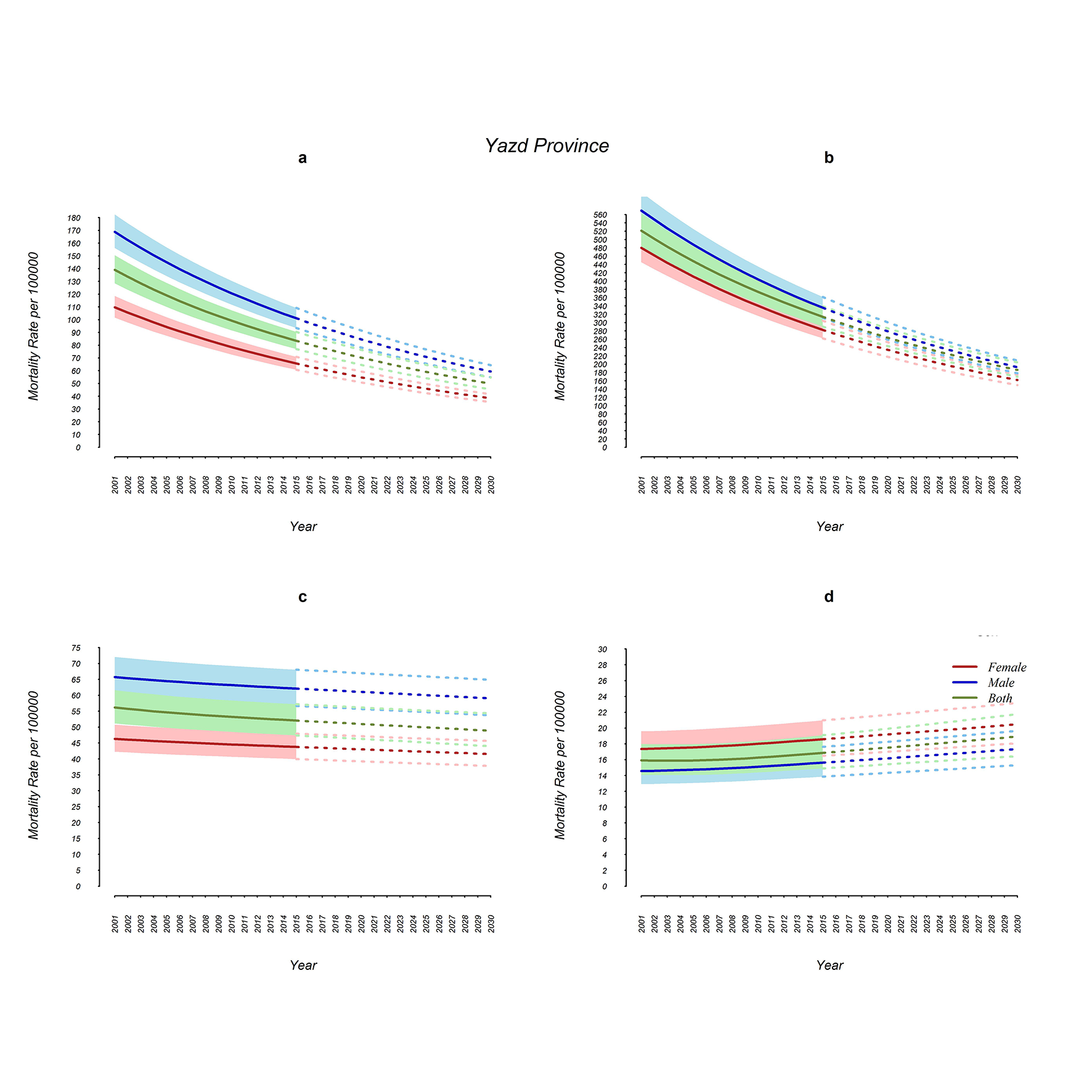

Supplement: S22 Fig — a) Cancer, b) CVDs, c) Asthma and COPD, d) Diabetes. Yazd province. (TIF) [file pone.0211622.s023.tif]

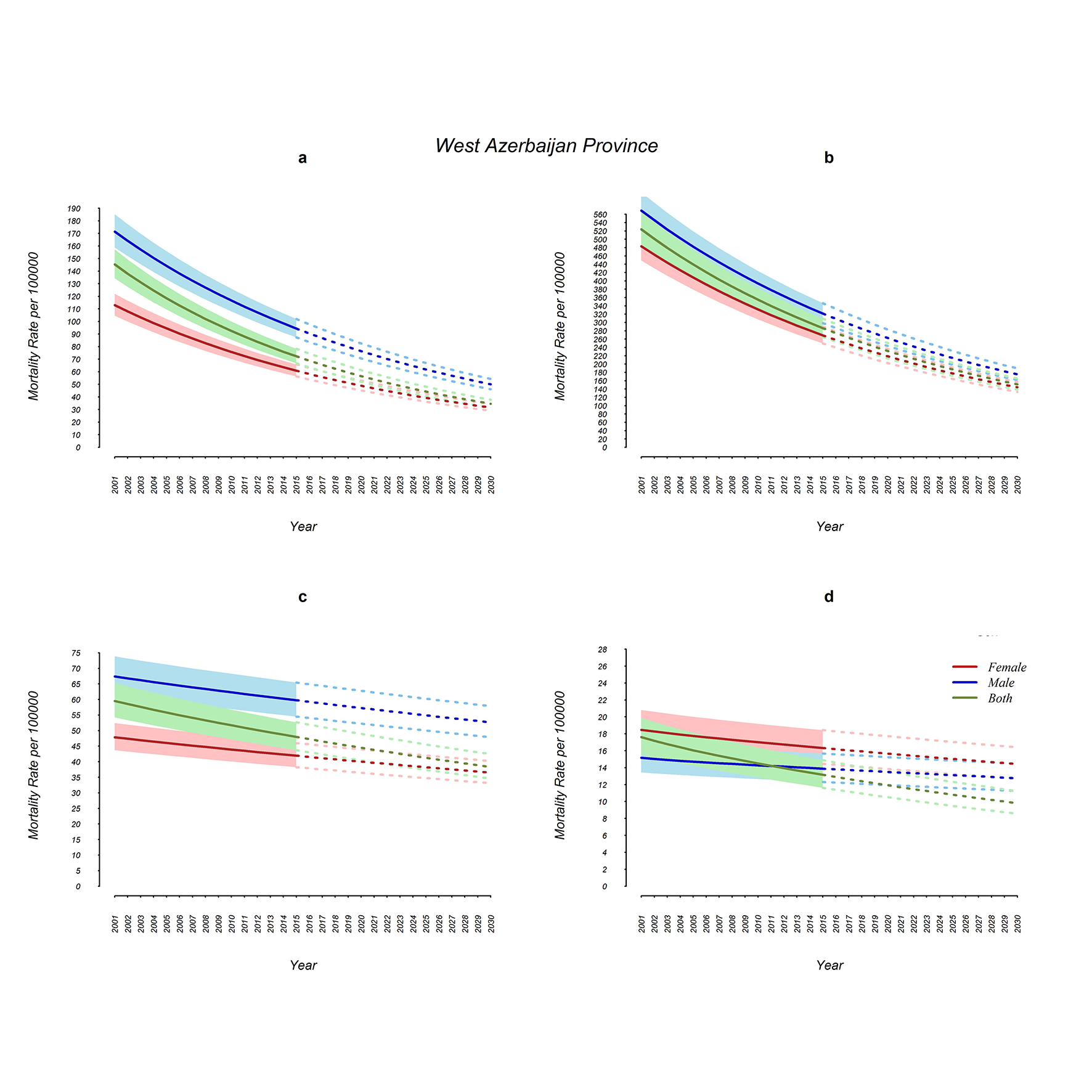

Supplement: S23 Fig — a) Cancer, b) CVDs, c) Asthma and COPD, d) Diabetes. West Azarbiajan province. (TIF) [file pone.0211622.s024.tif]

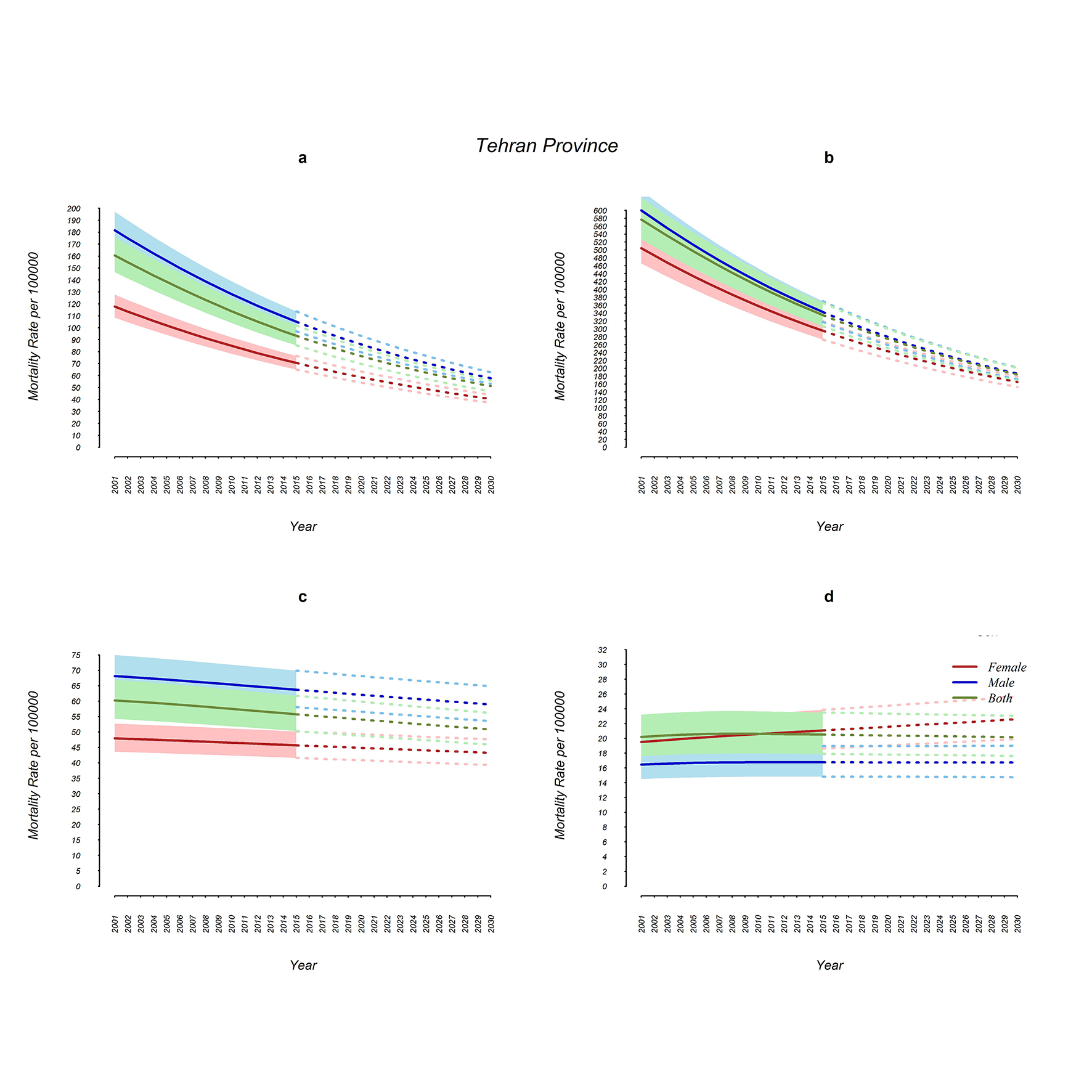

Supplement: S24 Fig — a) Cancer, b) CVDs, c) Asthma and COPD, d) Diabetes. Tehran province. (TIF) [file pone.0211622.s025.tif]

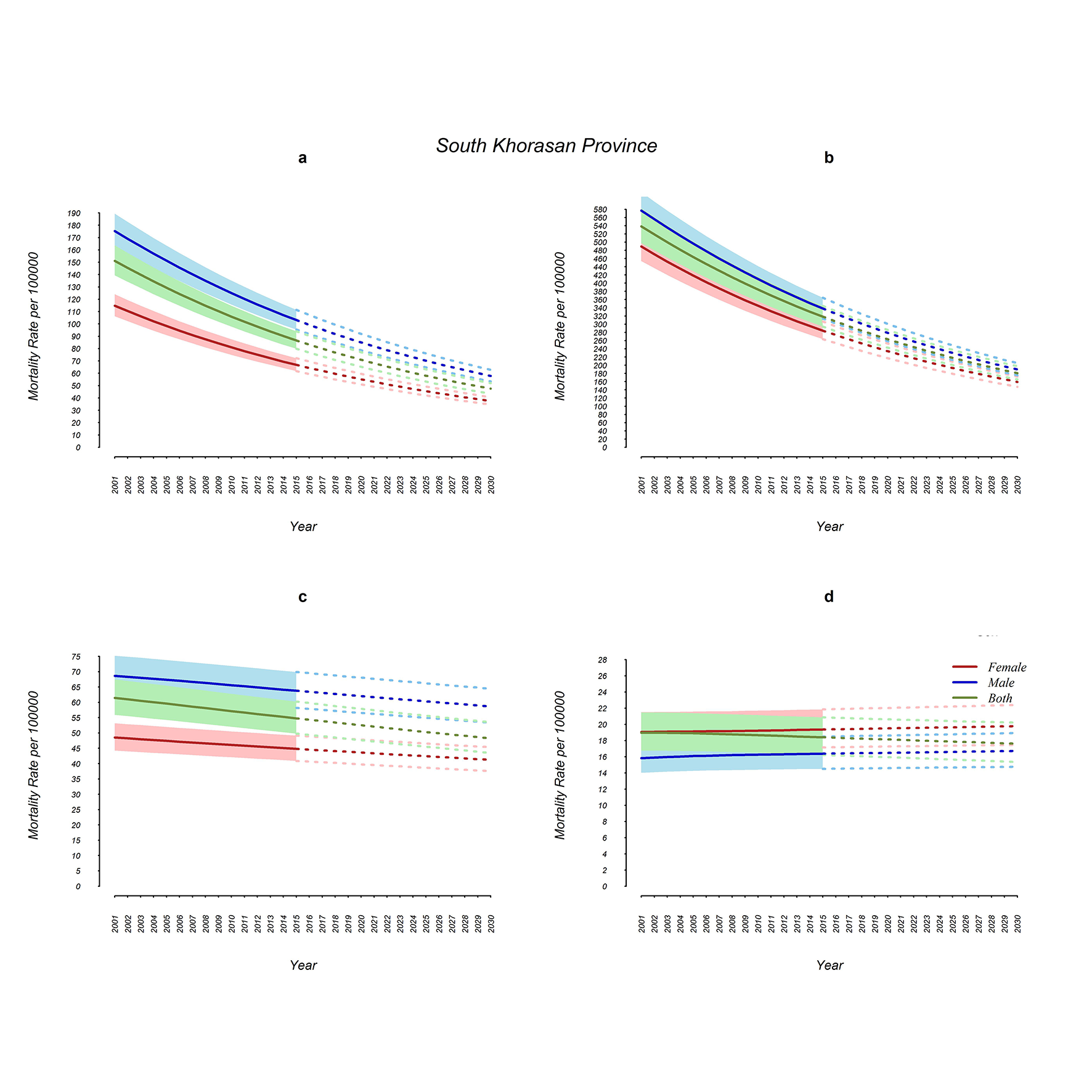

Supplement: S25 Fig — a) Cancer, b) CVDs, c) Asthma and COPD, d) Diabetes. South Khorasan province. (TIF) [file pone.0211622.s026.tif]

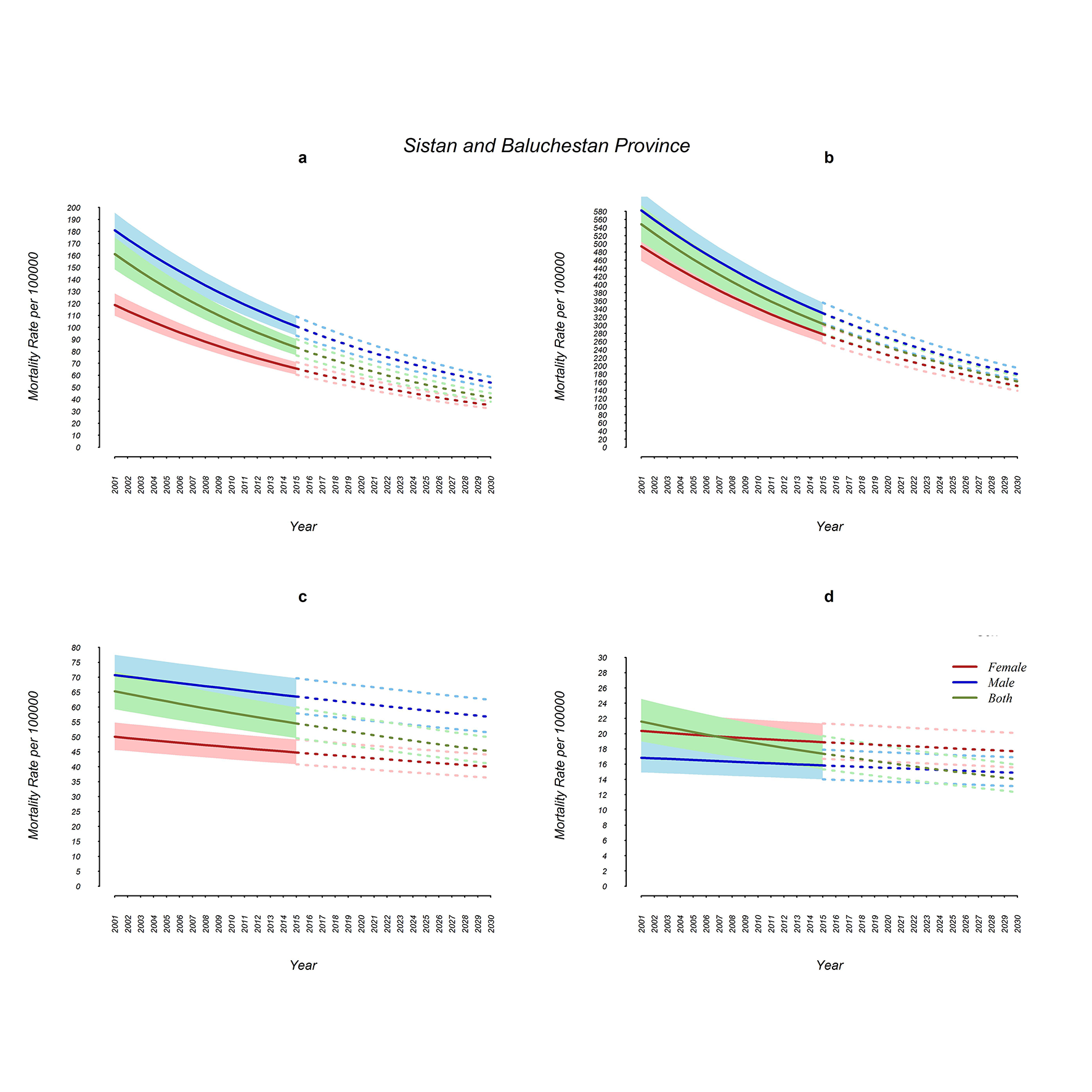

Supplement: S26 Fig — a) Cancer, b) CVDs, c) Asthma and COPD, d) Diabetes. Siatan and bluchestan province. (TIF) [file pone.0211622.s027.tif]

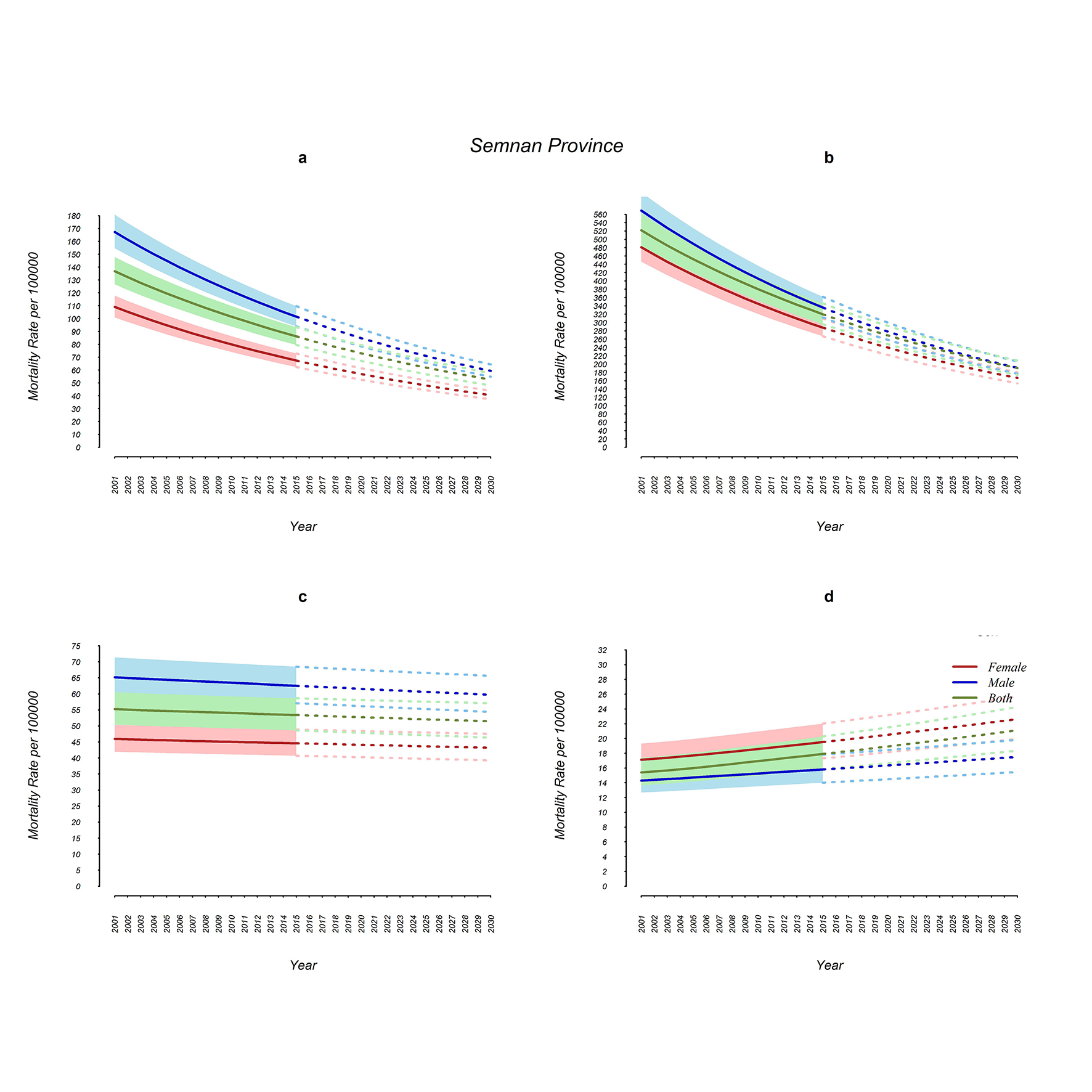

Supplement: S27 Fig — a) Cancer, b) CVDs, c) Asthma and COPD, d) Diabetes. Semnan province. (TIF) [file pone.0211622.s028.tif]

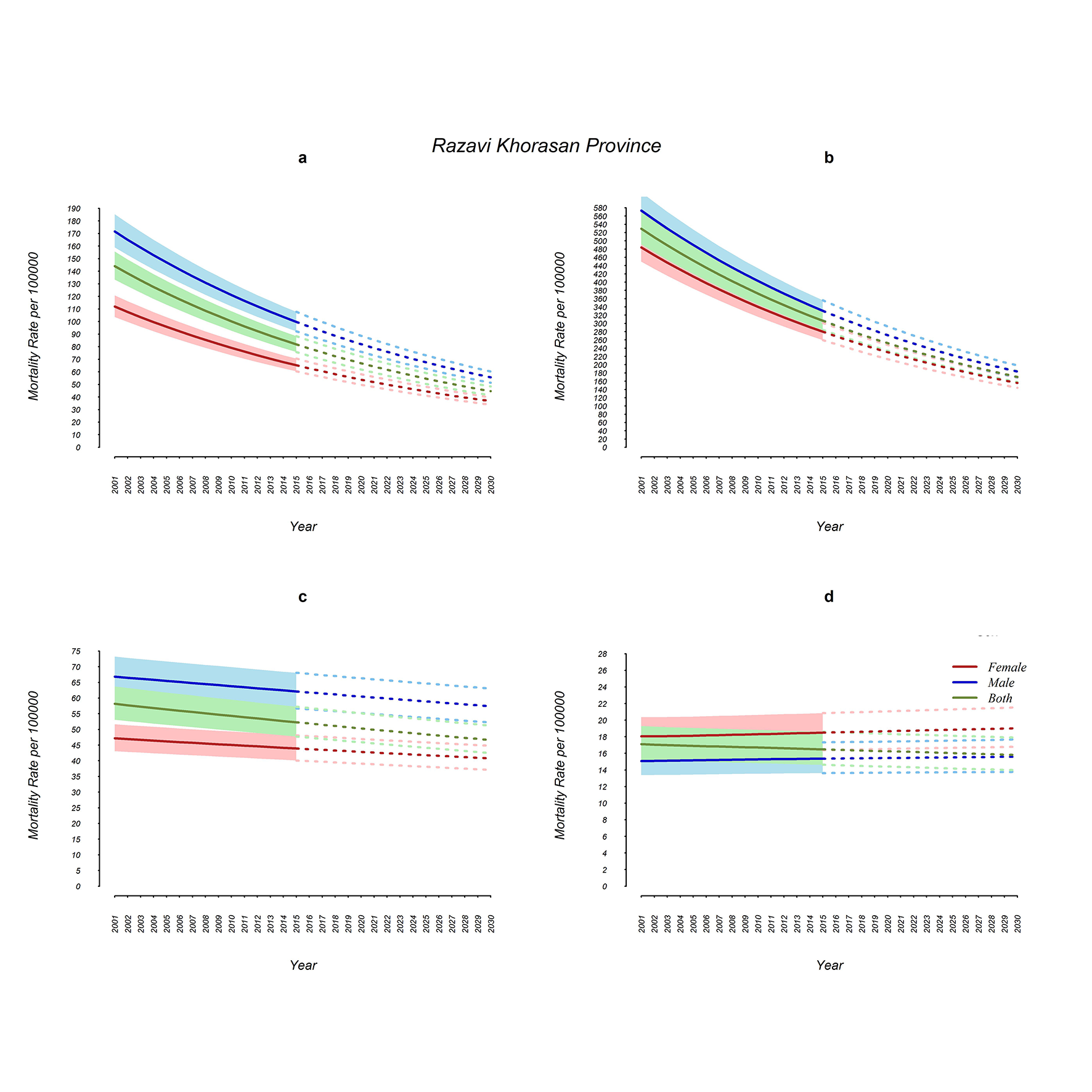

Supplement: S28 Fig — a) Cancer, b) CVDs, c) Asthma and COPD, d) Diabetes. Razavi Khorasan province. (TIF) [file pone.0211622.s029.tif]

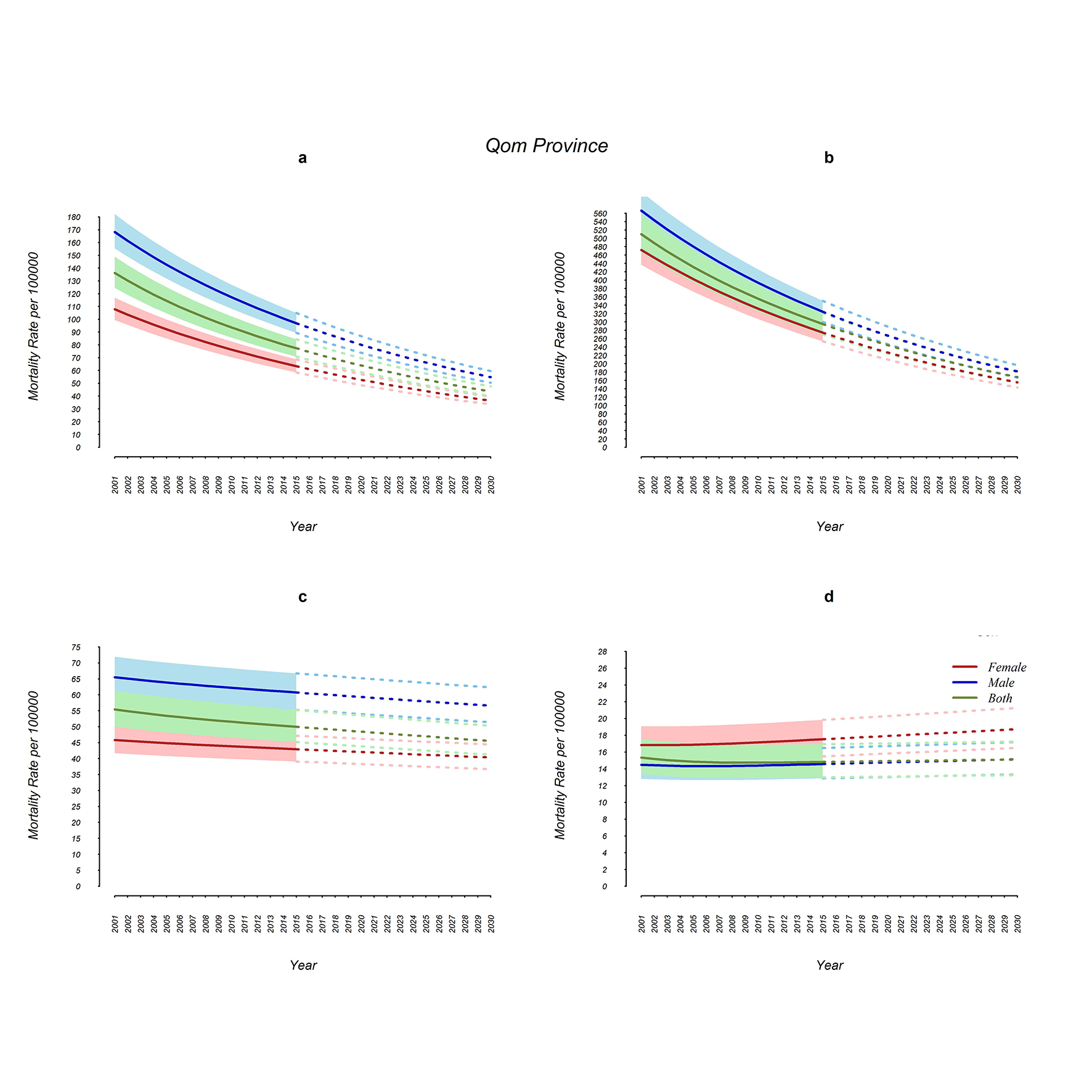

Supplement: S29 Fig — a) Cancer, b) CVDs, c) Asthma and COPD, d) Diabetes. Qom province. (TIF) [file pone.0211622.s030.tif]

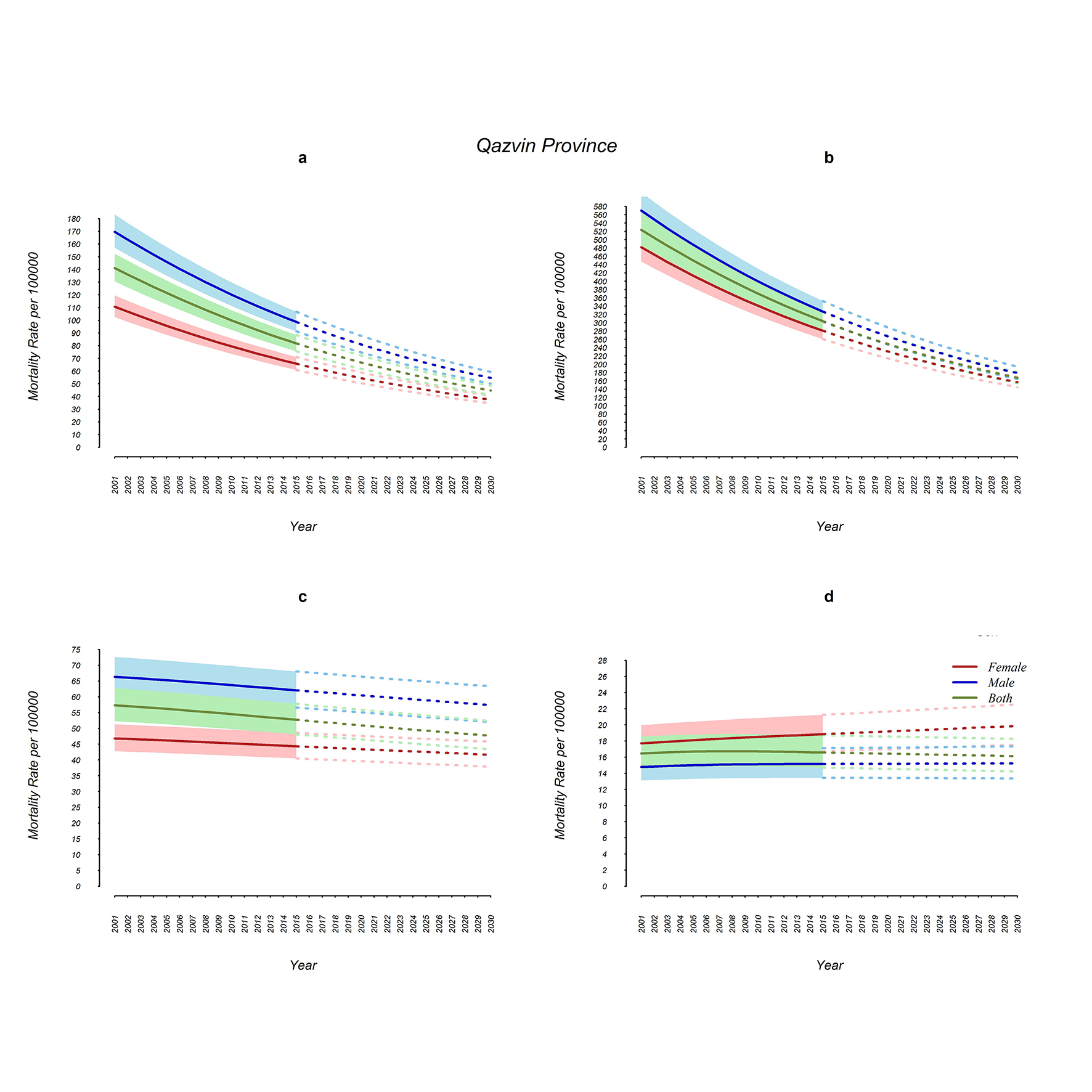

Supplement: S30 Fig — a) Cancer, b) CVDs, c) Asthma and COPD, d) Diabetes. Qazvin province. (TIF) [file pone.0211622.s031.tif]

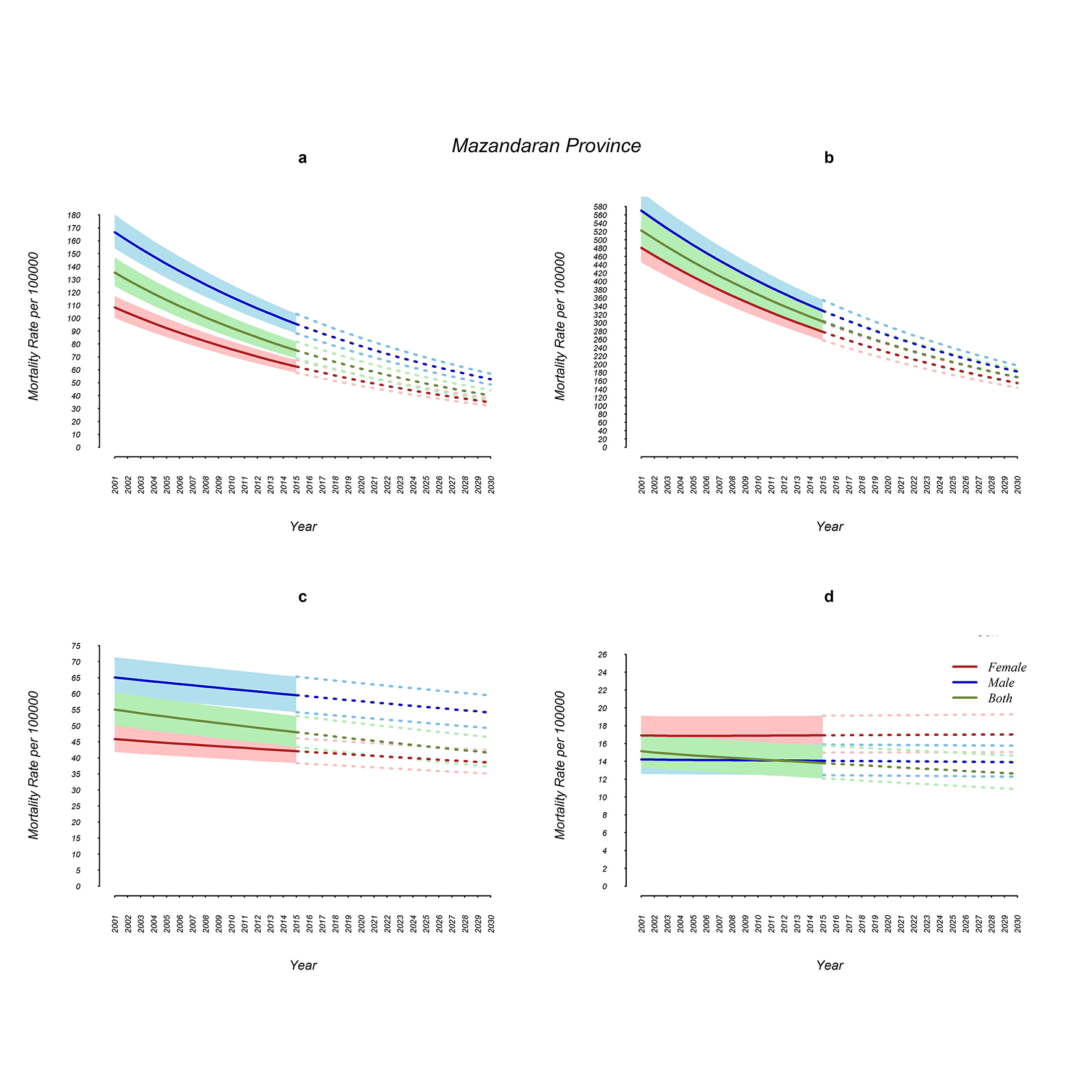

Supplement: S31 Fig — a) Cancer, b) CVDs, c) Asthma and COPD, d) Diabetes. Mazandaran province. (TIF) [file pone.0211622.s032.tif]

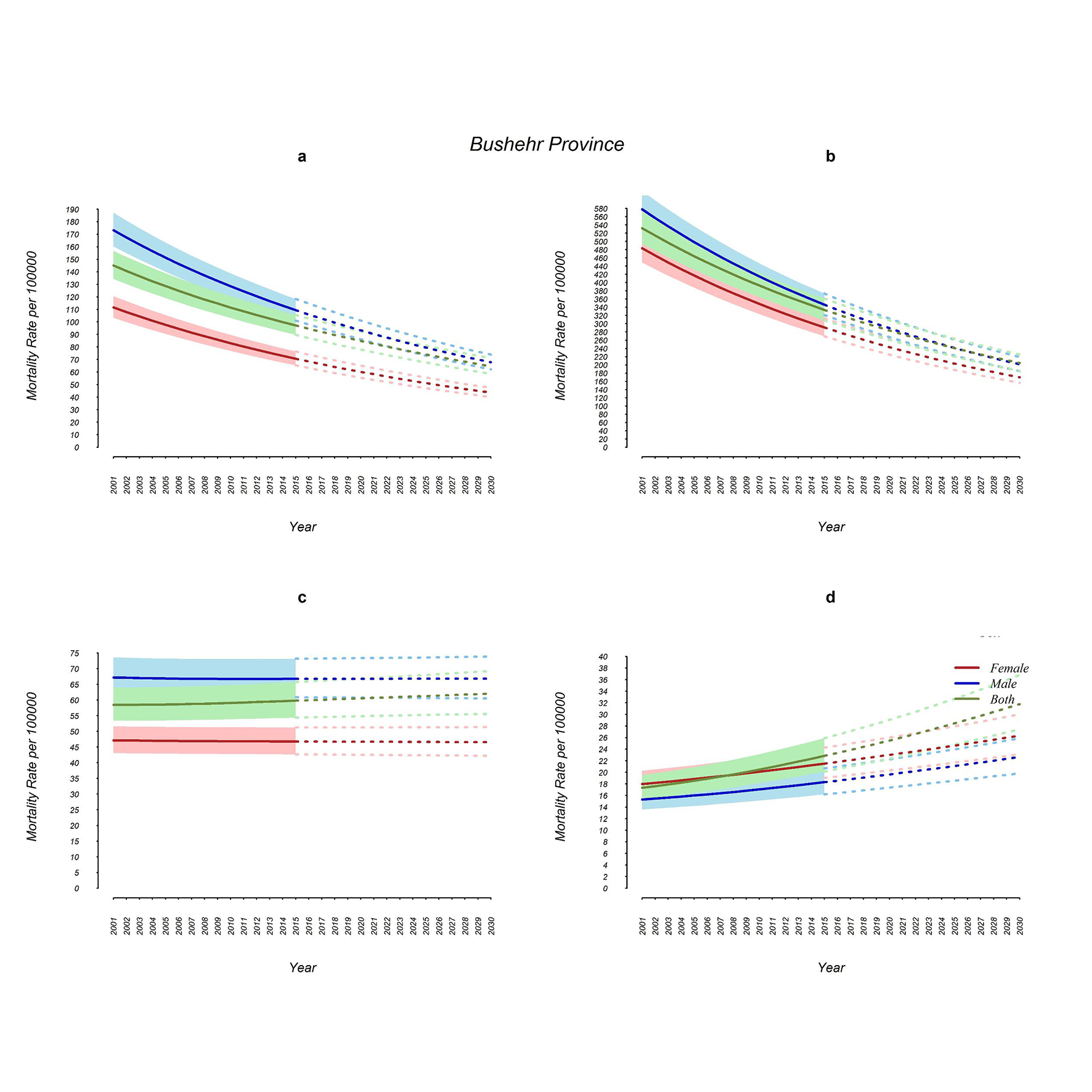

Supplement: S32 Fig — a) Cancer, b) CVDs, c) Asthma and COPD, d) Diabetes. Bushehr province. (TIF) [file pone.0211622.s033.tif]
